# Investigating the Crystallization Kinetics and Mechanical Properties of Poly(Butylene Succinate): The Dual Role of Polyoxymethylene Blending and MWCNT Heterogeneous Nucleation

**DOI:** 10.3390/polym18172125

**Published:** 2026-08-31

**Authors:** Cansın Tutuş, Rumeysa Yıldırım, Nazlı Yazıcı Çakır, Merve Gökmen, Güralp Özkoç, Mehmet Kodal

**Affiliations:** 1 Department of Chemical Engineering, Kocaeli University, 41001 Kocaeli, Türkiye; c.ussluel@gmail.com (C.T.); rumeysa.yildirim@kocaeli.edu.tr (R.Y.); nazli.yazici@kocaeli.edu.tr (N.Y.Ç.); gokmenmerve1@gmail.com (M.G.); 2 Department of Chemistry and Chemical Processing Technologies, Hereke Asım Kocabıyık Vocational School, Kocaeli University, 41800 Kocaeli, Türkiye; 3 Department of Machinery and Metal Technologies, Hereke Asım Kocabıyık Vocational School, Kocaeli University, 41800 Kocaeli, Türkiye; 4 Xplore Instruments B.V., 6135 KT Sittard, The Netherlands; guralp.ozkoc@istinye.edu.tr; 5 Department of Chemistry, Istinye University, 34396 Istanbul, Türkiye; 6 Polymer Science and Technology Graduate Program, Kocaeli University, 41001 Kocaeli, Türkiye

**Keywords:** PBS, POM, MWCNT, non-isothermal crystallization kinetics, mechanical properties

## Abstract

This study investigates the crystallization behavior of poly(butylene succinate) (PBS) through blending with highly crystalline polyoxymethylene (POM) to address the inherently slow crystallization rate of PBS. The non-isothermal crystallization kinetics of the individual constituents within the blend systems were systematically investigated to elucidate their crystallization behavior under cooling conditions. In addition, the influence of multi-walled carbon nanotube (MWCNT) incorporation on the mechanical, thermomechanical, and morphological properties of PBS/POM blends was evaluated. The crystallization behavior was analyzed using kinetic approaches under non-isothermal conditions. Spherulite morphology was observed via polarized optical microscopy equipped with a controlled heating–cooling stage to elucidate spherulitic development. Scanning electron microscopy (SEM) revealed no obvious micron-scale phase separation in the PBS/POM blends, while the distinct crystallization behavior of the PBS and POM phases observed by differential scanning calorimetry (DSC) indicated that the two components retained their individual crystalline phases, supporting the partial miscibility of the blends. In the ternary systems, SEM observations showed a relatively uniform distribution of MWCNTs at the spatial scale accessible by SEM, with no pronounced micron-scale agglomerates readily discernible within the examined regions. Mechanical analyses demonstrated that the incorporation of MWCNTs enhanced the properties of the blends, particularly the elastic modulus and tensile strength. Kinetic analysis further indicated that MWCNTs influenced the crystallization behavior of the PBS and POM phases through nucleation effects, with their influence depending on the PBS/POM blend composition.

## 1. Introduction

Most plastic products that are used in our everyday lives such as polypropylene (PP), polyethylene (PE), polystyrene (PS), poly(vinyl chloride) (PVC) and poly(ethylene terephthalate) (PET) are derived from petrochemical resources [1,2]. These polymers exhibit excellent mechanical properties, good thermal stability, and high chemical and biological inertness, making them widely applicable in the packaging industry, including the production of bottles, films, and plastic bags. However, these materials exhibit a high resistance to biodegradation, resulting in their prolonged persistence in the environment. Furthermore, difficulties in recycling and reuse, largely attributable to varying levels of contamination, have led to their accumulation as a significant component of municipal solid waste [1,3]. The extensive utilization of plastics has contributed to the growing accumulation of plastic waste, commonly termed “white pollution,” which represents a major environmental concern due to its persistence and resistance to degradation in both terrestrial and aquatic environments [4,5]. Packaging industries use up to 40% of produced plastics for short service life applications, and most of these plastics end up in landfills. There is thus a need to develop biodegradable polymers with similar functionality as petrochemical polymers, but that are readily susceptible to microbial action. This will contribute to a reduction in the environmental pollution caused by plastic waste and also conserve petrochemical resources [1,3,6,7,8]. Although global plastic production surpasses 368 million tons per year, bioplastics represent only approximately 1% of this total output [9]. At the same time, approximately 50–55% of these plastics are widely used for single-use and short-term purposes, with much of them being discarded indiscriminately or disposed of in landfills [5].

Biodegradable polymers are mainly synthesized from renewable natural resources, and they degrade over a period of time through enzymolysis of microorganisms when exposed to a natural environment [3]. Biodegradable and compostable polymers offer an alternative, because they can degrade in a defined period of time without having a negative environmental impact [5]. Biodegradable polymers can be broadly classified into three main categories according to their origin and production route. The first category comprises natural biodegradable polymers obtained from renewable resources, including starch, cellulose, lignin, chitosan, and various proteins. The second category consists of polyhydroxyalkanoates (PHAs), a family of biodegradable polyesters biosynthesized by bacteria and other microorganisms. The third category includes synthetic biodegradable polymers produced through the polymerization of monomers derived from either renewable or petrochemical feedstocks. Representative polymers within this group include poly(lactic acid) (PLA), polycaprolactone (PCL), poly(butylene succinate) (PBS), and poly(butylene adipate-*co*-terephthalate) (PBAT), all of which have attracted considerable interest for biodegradable material applications [9].

Among the biodegradable polymers currently under investigation, PBS is regarded as one of the most promising. PBS is an aliphatic polyester produced through the polycondensation of succinic acid and 1,4-butanediol, which may be obtained from renewable feedstocks. Notably, succinic acid can be generated through the microbial fermentation of biomass-derived substrates, including sugars, glucose, starch, and xylose [10,11]. Compared with other aliphatic polyesters, PBS exhibits favorable biodegradability, melt processability, thermal properties, and chemical resistance. These characteristics make PBS a promising material for a wide range of plastic applications, including the manufacture of injection-molded products, films, and fibers [12,13,14,15]. In addition, PBS exhibits mechanical properties comparable to those of conventional polyolefins, including relatively high tensile strength and moderate hardness and stiffness [11]. Owing to these advantages, PBS has been widely applied in areas such as packaging materials, agricultural films, and biomedical applications. However, its relatively low stiffness, low melt viscosity, and slow crystallization rate limit its processability and broader application potential, particularly in injection molding. Specifically, the low melt viscosity is not ideal for processing requirements, while in film and fiber production, crystallization can be enhanced through drawing. In contrast, such orientation-induced crystallization cannot be achieved in injection molding. Consequently, slow crystallization leads to prolonged molding cycles and increased processing costs. Therefore, improving the performance of PBS is of considerable importance. Various approaches, including blending with other polymers, chemical copolymerization, and nanocomposite formation, have been investigated to enhance its properties [12,16,17,18,19].

Within this framework, incorporating polyoxymethylene (POM), a highly crystalline engineering thermoplastic, into polymer blends has garnered significant attention as a viable strategy to enhance the performance of biodegradable polymers. POM is a linear thermoplastic synthesized from formaldehyde and is characterized by a highly regular molecular structure with no pendant side groups, resulting in high crystallinity and density. Due to this distinct molecular architecture, POM exhibits an exceptional balance of properties, including superior tensile and flexural strength, outstanding chemical and creep resistance, high dimensional stability, and excellent tribological behavior (low friction and wear resistance). Consequently, POM serves as a critical engineering material widely deployed in high-precision components, such as mechanical bearings, transmission gears, irrigation systems, and structural pulleys [20]. Simultaneously, driven by its high thermodynamic driving force for crystallization, POM undergoes rapid phase transformation to form well-defined spherulites. This characteristic is exploited in polymer blending to promote the crystallization kinetics of inherently slow-crystallizing polymers. In a representative study, Jiao et al. systematically elucidated the miscibility, crystallization mechanisms, and mechanical performance of melt-processed POM/PBS blends. Owing to the higher melting point of the POM phase, it crystallizes preferentially, forming a rigid spherulitic crystalline network; the PBS phase subsequently crystallizes within the restricted interfibrillar regions of this pre-existing POM skeleton. Crucially, the POM crystalline surfaces function as efficient heterogeneous nucleation sites, accelerating PBS crystallization at optimized blend ratios. From a mechanical standpoint, blending with PBS suppresses the brittleness of POM while enhancing its impact toughness. Moreover, the robust thermal stability of the continuous POM crystalline network drastically enhances both the thermal stability and upper service temperature limit of PBS [21]. In a related study, POM was utilized as a macromolecular nucleating agent to accelerate the inherently lower crystallization kinetics of poly(lactic acid) (PLA) while preserving its optical transparency. PLA/POM blends were melt-compounded via twin-screw extruder, followed by an extensive characterization of their crystallization behavior and crystalline morphology. The findings demonstrated that the incorporation of POM significantly promoted PLA crystallization. Specifically, increasing the POM fraction induced a downward shift in the cold crystallization temperature, widened the disparity between the melting and crystallization enthalpies, and elevated the melt crystallization temperature. Furthermore, polarized optical microscopy observations revealed a systematic reduction in spherulite dimensions alongside a concomitant increase in nucleation density as a function of POM content [22].

The properties of polymer blends can be further improved through the addition of nanofillers to obtain blend-based nanocomposites. Indeed, the distribution of fillers and the interfacial interaction between polymers and fillers play key roles in controlling the final properties of nanocomposites. Fillers such as metal oxides, calcium sulphate whiskers, organoclays and carbon nanotubes (CNTs) have been used to reinforce polymer blends [16,23,24,25,26]. These fillers not only provide substantial mechanical reinforcement to the blends but also significantly enhanced the interfacial adhesion between the constituent polymer phases [23]. On the other hand, it has been proven that adding nanoparticles into such blends could influence the crystallization processes. In particular, nanoparticles are usually good nucleating agents, and they can enhance the nucleation ability of a polymer matrix, exhibiting an increased nucleation sites and crystallization rate. Herein, investigations on the effects of nanoparticles on the crystallization behaviors of thermodynamically immiscible blends with one or two crystallizable polymer components have attracted great interest [27]. Carbon nanotubes (CNTs) represent high-aspect-ratio allotropes of carbon atoms arranged in a cylindrical hexagonal lattice. These nanostructures, characterized by exceptional tensile strength and elastic modulus, are fundamentally classified into two variants: single-walled carbon nanotubes (SWCNTs) and multi-walled carbon nanotubes (MWCNTs). SWCNTs comprise a single graphene sheet seamlessly rolled into a hollow cylinder, whereas MWCNTs consist of multiple concentric, coaxial graphene cylinders surrounding a central hollow core, stabilized by interlamellar Van der Waals interactions. MWCNTs represent a more viable selection for large-scale applications; this preference is driven by their robust bulk availability and enhanced distribution efficiency compared to their single-walled counterparts [1,28,29].

The role of carbon nanotubes (CNTs) in modulating the crystallization kinetics of polymeric matrices is well documented in the literature. For instance, Barrau et al. comprehensively elucidated the influence of CNTs on the crystallization behavior of PLA. Differential scanning calorimetry (DSC) analyses demonstrated that the incorporation of CNTs significantly accelerated PLA crystallization kinetics. While neat PLA underwent crystallization exclusively at low cooling rates, the CNT-reinforced composites exhibited robust crystal development even under rapid cooling conditions. Isothermal crystallization kinetics revealed that a 1 wt% CNT loading reduced the total crystallization time by approximately 50%, which was accompanied by a sharp decrease in the crystallization half-time (t_1/2_). Furthermore, Avrami analysis indicated that the presence of CNTs primarily altered the nucleation mechanism rather than the crystal growth rate; this effective heterogeneous nucleation was corroborated by a shortened crystallization induction time and a concurrent increase in the Avrami kinetic constant. Polarized optical microscopy (POM) observations showed the formation of large spherulites with diameters of 3–5 μm in neat PLA, whereas CNT-containing composites exhibited much smaller and more densely distributed spherulitic structures. These findings demonstrate that CNT surfaces provide numerous nucleation sites, thereby increasing the number of crystallization nuclei and accelerating the overall crystallization rate [30]. In another study, Pramoda et al. prepared PBS/MWCNT nanocomposites containing various concentrations of MWCNTs via melt blending and investigated the effects of these nanofillers on crystallization kinetics, crystal morphology, and mechanical properties. The results revealed that the MWCNTs acted as highly potent heterogeneous nucleation centers within the PBS. As a consequence of the increased nucleation density, the spherulite size decreased significantly in the nanocomposites compared with neat PBS. Moreover, crystallization commenced at higher temperatures in the presence of MWCNTs. The crystallization peak temperature increased from approximately 83 °C for neat PBS to nearly 95 °C with MWCNT incorporation, while the crystallization half-time decreased considerably, indicating a pronounced increase in crystallization rate. In addition to enhancing crystallization behavior, the addition of only 0.1 wt% MWCNT resulted in an approximately 23% increase in storage modulus, demonstrating a notable improvement in mechanical performance [31]. Similarly, Zhao and Ye investigated the effects of poly(ethylene glycol) (PEG)-functionalized MWCNTs on the mechanical properties and crystallization behavior of POM. DSC analysis revealed that MWCNTs served as highly efficient heterogeneous nucleation centers for POM. The addition of MWCNTs increased both the crystallization onset temperature and crystallization peak temperature, reduced the degree of supercooling, and narrowed the crystallization exotherms, indicating accelerated crystallization kinetics. Isothermal crystallization analyses further showed that MWCNT incorporation shortened the crystallization induction time and increased the Avrami kinetic constant (k), resulting in a lower crystallization half-time. These results suggest a substantial enhancement in crystal growth and crystallization rate. POM observations revealed that neat POM formed large and well-defined spherulites, whereas MWCNT-containing composites exhibited significantly smaller spherulites and finer crystalline structures due to the formation of numerous nucleation sites. Furthermore, crystal formation was observed in the composites even at temperatures where neat POM was unable to crystallize. This enhanced crystallization behavior led to simultaneous improvements in tensile strength, elongation at break, and impact resistance. The authors concluded that MWCNTs significantly promote crystallization within the POM matrix, leading to a finer and more homogeneous crystalline morphology and consequently improving both the stiffness and toughness of POM [32].

The primary objective of this study is to systematically investigate the effects of blending poly(butylene succinate) (PBS) with polyoxymethylene (POM), a highly crystalline engineering thermoplastic, and incorporating multi-walled carbon nanotube (MWCNT) reinforcement to overcome the inherent drawbacks of PBS, such as slow crystallization rates, which restrict its injection molding processability and industrial applications. While the existing literature extensively covers the crystallization kinetics of single-polymer matrices, this research offers a unique novelty by providing a deep-dive analysis of the non-isothermal crystallization kinetics of PBS/POM binary blend systems utilizing the Avrami, Ozawa and combined Avrami–Ozawa models, alongside the simultaneous visualization of morphological evolution through temperature-controlled polarized optical microscopy (POM). Activation energies for crystallization of PBS and its nanocomposites were evaluated by the Friedman model. The crystallographic characteristics and degree of crystallinity of the samples were determined by X-ray diffraction (XRD) analysis. Furthermore, a comprehensive investigation of the influence of MWCNT distribution on the mechanical, thermomechanical, and microstructural properties significantly enhances the novelty and distinctiveness of this study. Additionally, elucidating the influence of MWCNT distribution at different loading levels on the crystallization behavior through variations in crystallization temperatures and validating the compatibility between the PBS and POM using FTIR and SEM analyses provide an original and pioneering contribution to the literature on the performance optimization of bio-based/engineering polymer blends.

## 2. Materials and Methods

### 2.1. Materials

Poly(butylene succinate) (PBS, BioPBS™ FZ71 (PM/PB)) was supplied by Mitsubishi Chemical Co. (Tokyo, Japan) and exhibited a melting temperature in the range of 90–120 °C. It has a density of 1.26 g/cm^3^ and a melt flow rate (MFR) of 22 g/10 min (190 °C/2.16 kg). Polyoxymethylene (POM, Delrin^®^ 100T NC010) was purchased from Delrin, Wilmington, DE, USA, and possessed a crystallinity of approximately 70–80%. The MFR value of POM is 2.1 g/10 min at 190 °C under a 2.16 kg load, and the density is 1.37 g/cm^3^. The pristine multi-walled carbon nanotubes (MWCNTs, NG01MW0314), with an outside diameter of 8–10 nm, an average length of 1–3 µm, a purity exceeding 90%, and a specific surface area of 290 m^2^/g, were obtained from Nanografi (Ankara, Türkiye).

### 2.2. Processing

Prior to melt compounding, PBS and POM pellets were dried in a vacuum oven at 80 °C for 12 h to remove residual moisture. Melt blending was subsequently carried out using a laboratory-scale co-rotating twin-screw micro-compounder (MC 15 HT, Xplore Instruments B.V., Sittard, The Netherlands) with a chamber volume of 15 mL. The compounding process was performed at a barrel temperature of 190 °C, a screw speed of 100 rpm, and a residence time of 3 min. To suppress thermo-oxidative degradation during processing, nitrogen was continuously purged through the barrel throughout the melt-blending process.

Following melt compounding, the blends were injection molded using a laboratory-scale micro-injection molding machine (IM 12, Xplore Instruments B.V., The Netherlands) with a 12 mL injection capacity to produce standard test specimens conforming to ISO 527-2/5A and ISO 180 specifications. The injection pressure was maintained at 10 bar, while the melt and mold temperatures were set to 190 °C and 25 °C, respectively.

Melt-compounded formulations containing 0.5 and 1.0 wt.% MWCNTs were prepared based on neat PBS, neat POM, and PBS/POM blends. The blend compositions consisted of PBS/POM weight ratios of 75/25 and 50/50. The naming of the samples was based on the concentration of the materials they contained. For example, the sample 75PBS/25POM/1MWCNT consists of 75% PBS, 25% POM, and 1% MWCNT by weight, respectively.

### 2.3. Characterization

The molecular interactions among PBS, POM, and MWCNTs were investigated using Fourier transform infrared (FTIR) spectroscopy with a PerkinElmer Spectrum 100 spectrometer (PerkinElmer, Waltham, MA, USA) equipped with a diamond attenuated total reflectance (ATR) accessory. Spectra were acquired over the wavenumber range of 4000–650 cm^−1^.

The morphological properties of the samples were examined using a JEOL JIB-450 scanning electron microscope (SEM) (JEOL Ltd., Akishima, Tokyo, Japan). SEM images were obtained from the cryogenically fractured surfaces of impact-tested specimens to evaluate the fracture morphology. Before SEM observation, the samples were sputter-coated with a thin layer of gold to enhance surface conductivity and prevent charging effects during imaging.

The mechanical properties of the samples were evaluated using tensile and Izod impact tests. Tensile tests were conducted in accordance with ISO 527-5A using an Instron Model 3345 universal testing machine (Instron, Norwood, MA, USA) at a crosshead speed of 10 mm/min. Izod impact strength measurements were performed on 2 mm V-notched specimens using a Ceast Resil Impactor (CEAST, Pianezza, Turin, Italy), in accordance with the ISO 180 standard.

The viscoelastic properties of the samples were investigated using a PerkinElmer Pyris Diamond dynamic mechanical analyzer (DMA) (PerkinElmer, Waltham, MA, USA). The measurements were conducted at a heating rate of 3 °C/min and a frequency of 1 Hz over a temperature range of −100 °C to 50 °C.

Differential scanning calorimetry (DSC, Mettler Toledo DSC 1 Star, Greifensee, Switzerland) was employed to investigate the non-isothermal crystallization kinetics of neat polymers, PBS/POM blends, and PBS/POM/MWCNT nanocomposites. Each DSC experiment consisted of two stages. In the first stage, the samples were heated from 25 °C to 200 °C at a heating rate of 15 °C/min and held isothermally for 5 min to erase the thermal history. Subsequently, in the second stage, the samples were cooled to 25 °C at different cooling rates of 5, 15, 25, and 50 °C/min. The kinetic parameters were derived from the cooling part of the DSC thermograms.

A polarized optical microscope (Nikon LV100 POL) (Nikon Corporation, Tokyo, Japan) equipped with a Linkam THMS600 hot stage was used to investigate crystal nucleation and growth in neat polymers, PBS/POM blends, and PBS/POM/MWCNT nanocomposites. The samples were placed between two glass slides and heated from 25 °C to 200 °C at a rate of 30 °C/min. To eliminate thermal history, the samples were held at 200 °C for 5 min and subsequently cooled to 30 °C at a cooling rate of 1 °C/min.

To identify the crystalline structures of neat PBS, neat POM, and PBS/POM blends with and without MWCNTs, X-ray diffraction (XRD) analysis was performed using a Malvern PANalytical Empyrean MultiCore diffractometer (Malvern Panalytical B.V., Almelo, The Netherlands) equipped with monochromatic CuKα radiation (λ = 1.54059 Å). The degree of crystallinity (*X_c_%*) was estimated by deconvoluting the diffraction patterns into their crystalline and amorphous contributions, followed by integration of the corresponding peak areas. Peak deconvolution and area integration were carried out using OriginPro 9.0 software, and the crystallinity percentage was calculated according to Equation (1):

(1) $$X_{c}\%=\frac{A_{c}}{A_{c}+A_{a}}\;*\;100$$

where *A_c_* and *A_a_* are the area under crystalline peaks and amorphous haloes, respectively.

Selective localization analyses were conducted to determine the preferential location of MWCNT nanoparticles within the PBS/POM blends. Contact angle measurements were employed to evaluate the localization tendency of the nanoparticles in the polymer matrix. The total surface free energy of a non-metallic material ($\gamma_{i}^{TOT}$) is composed of two components: the Lifshitz–van der Waals component ($\gamma_{i}^{LW}$) and the acid–base component ($\gamma_{i}^{AB}$). The methodology used to calculate these surface energy components has been described in detail in our previous study [33,34]. The surface energy components of the probe liquids employed in this study (diiodomethane, ethylene glycol, and formamide) are summarized in Table 1.

Static contact angle measurements were performed using a KSV Attention Theta goniometer. The measured contact angles were used to determine the polymer–filler and polymer–polymer interfacial tensions, from which the corresponding wettability parameters were calculated. To ensure the reliability and reproducibility of the measurements, at least five independent measurements were performed for each sample.

## 3. Results and Discussions

### 3.1. FTIR Results

**Figure 1:**
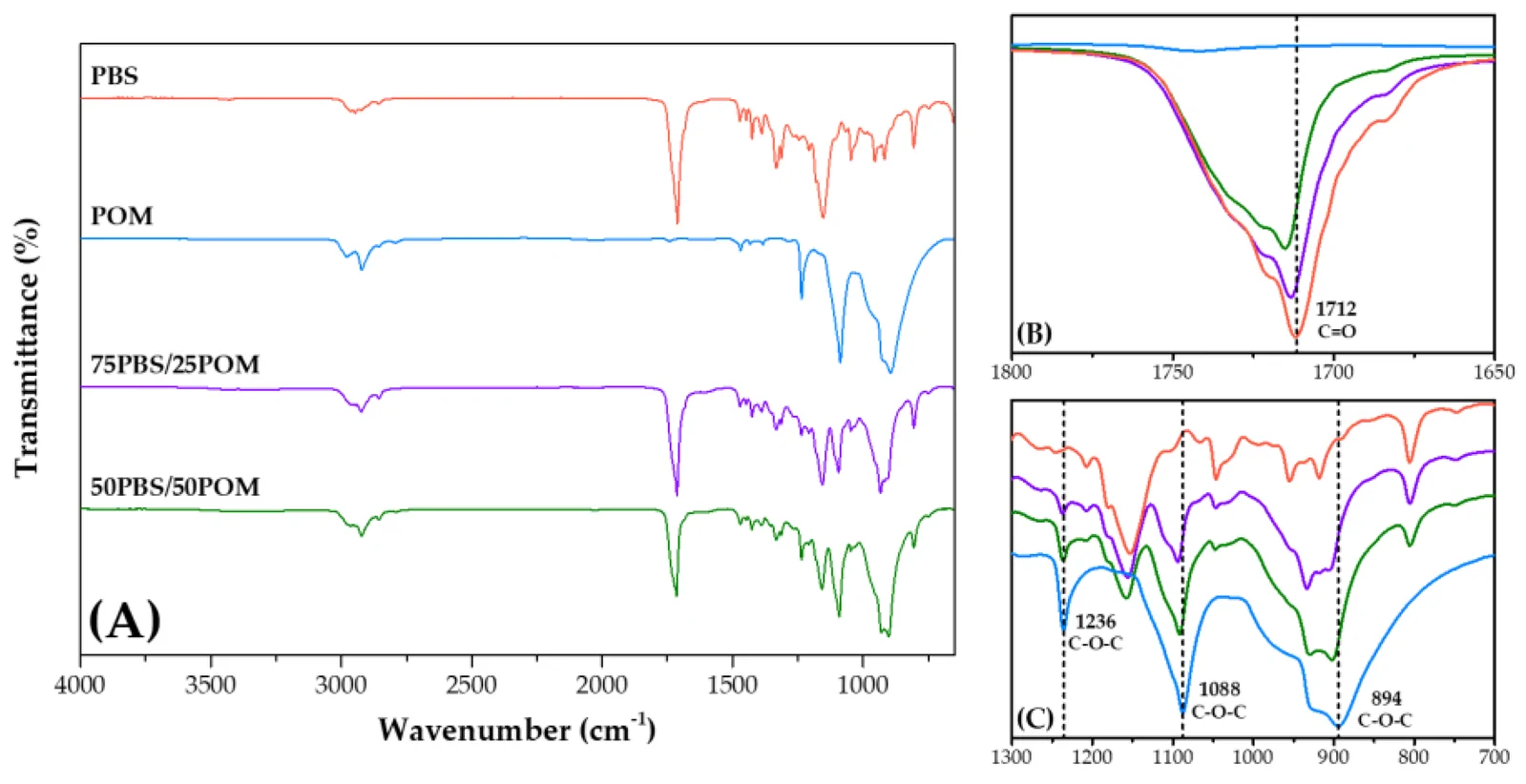
(**A**) FTIR spectra of PBS, POM and PBS/POM blends; (**B**) 1800–1650 cm^−1^ region; (**C**) 1300–700 cm^−1^ region.

Figure 1A presents the FTIR spectra of pure polymers (PBS and POM), PBS/POM blends, and their corresponding individual components over the wavenumber range of 4000 to 650 cm^−1^.

In the FTIR spectrum of PBS, the absorption bands observed at 2945 and 2854 cm^−1^ are assigned to the asymmetric and symmetric stretching vibrations of the –CH_2_– groups within the PBS main chain. The band appearing in the 1713–1710 cm^−1^ region is attributed to the C=O stretching vibration of the ester functionalities. The bands located in the 1264–1144 cm^−1^ region are associated with C–O–C stretching vibrations of the ester linkages, while those in the 1046–1044 cm^−1^ range are attributed to –O–C–C– stretching modes [36,37]. For POM, the characteristic absorptions at 2980 and 2922 cm^−1^ correspond to the asymmetric and symmetric stretching vibrations of C–H bonds, respectively, whereas the bands at 1469 and 1383 cm^−1^ are assigned to C–H bending vibrations. The appearance of bands at 1236, 1088, and 894 cm^−1^ is attributed to C–O–C vibrational modes, including bending, symmetric, and asymmetric stretching vibrations, respectively [38,39]. In the FTIR spectra of PBS/POM blends, the carbonyl (-C=O) stretching band of PBS, originally observed at 1712 cm^−1^, was shifted to 1714 and 1716 cm^−1^ upon incorporation of 25 wt% and 50 wt% POM, respectively (Figure 1B). In addition, the C–O–C-related bands of POM at 1236, 1088, and 894 cm^−1^ exhibited shifts toward lower wavenumbers in blends containing 25 wt% and 50 wt% POM (Figure 1C). These systematic peak shifts (<4 cm^−1^), which fall within the typical instrumental resolution of ATR-FTIR, can be attributed to physical interactions between the carbonyl groups of PBS and the ether (C–O–C) linkages of the POM chains during melt blending, together with the effects of physical dispersion, dilution, and local molecular interactions arising from processing.

**Figure 2:**
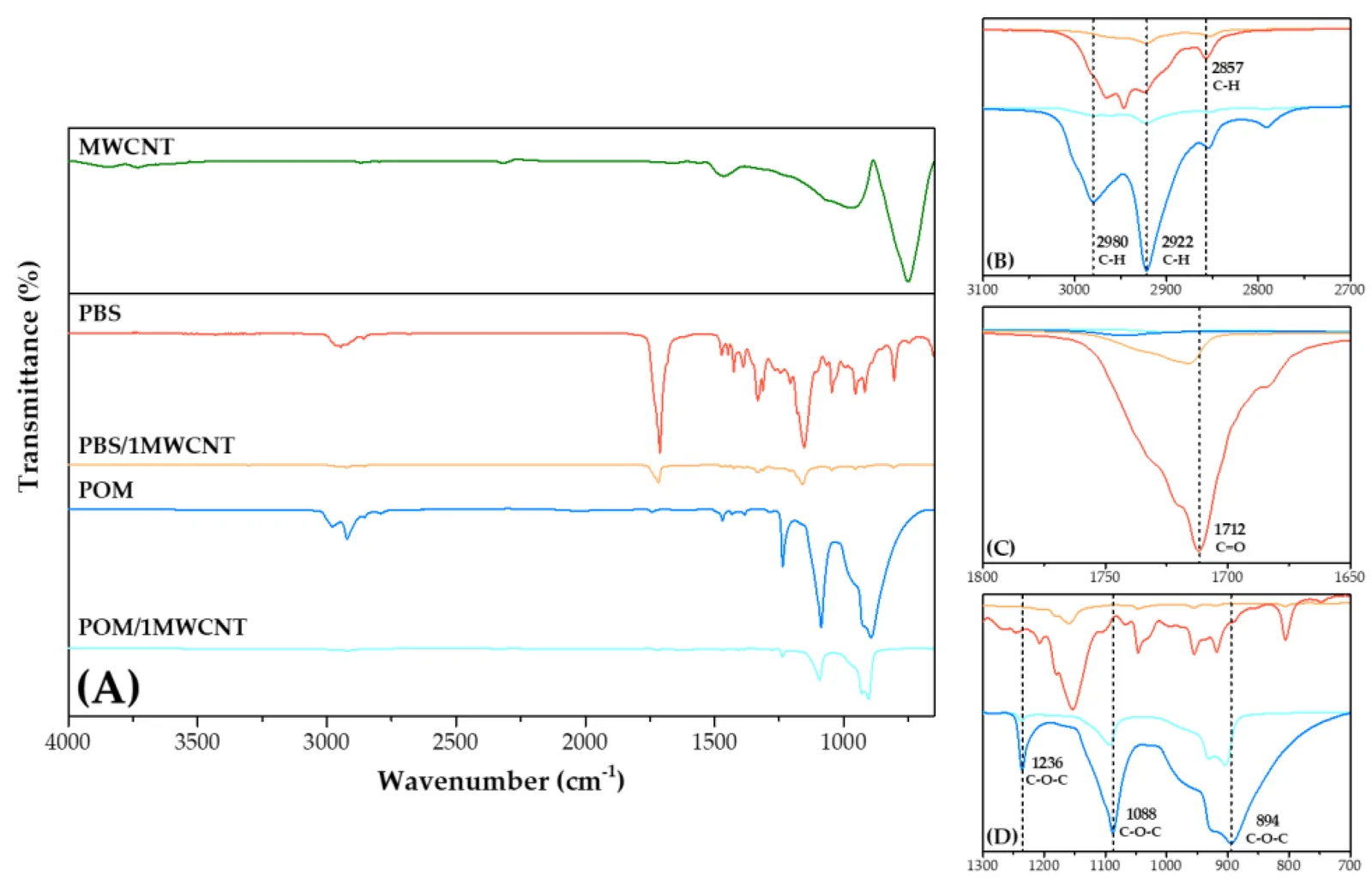
(**A**) FTIR spectra of MWCNT, PBS, POM, PBS/1MWCNT and POM/1MWCNT nanocomposites; (**B**) 3100–2700 cm^−1^ region; (**C**) 1800–1650 cm^−1^ region; (**D**) 1300–700 cm^−1^ region.

Figure 2A displays the FTIR spectra of MWCNT, PBS, POM, PBS/1MWCNT and POM/1MWCNT samples. The absorption bands observed in the FTIR spectrum of MWCNTs within the 15801530 cm^−1^ and 1200–1100 cm^−1^ regions are assigned to C=C stretching vibrations, whereas the band appearing around 850 cm^−1^ is attributed to vibrations associated with the C–C aromatic skeletal structure of the carbon framework [40,41]. The incorporation of MWCNTs into PBS and/or POM resulted in small changes in the FTIR spectra of the neat polymers. The C–H stretching vibration bands of POM at 2980 and 2922 cm^−1^, as well as that of PBS at 2857 cm^−1^, shifted to 2979, 2920, and 2852 cm^−1^, respectively, upon MWCNT addition, accompanied by a reduction in peak intensities (Figure 2B). These small spectral changes may be associated with changes in the local molecular environment of the polymer chains in the vicinity of the MWCNT surfaces. Although MWCNTs do not contain reactive functional groups capable of readily forming covalent bonds with PBS and/or POM, the observed changes could be consistent with weak non-covalent interactions, including possible interactions between polymer C–H groups and the π-electron-rich surface of MWCNTs [42,43]. However, the magnitude of these shifts is relatively small and, therefore, does not provide definitive evidence for a specific type of intermolecular interaction.

**Figure 3:**
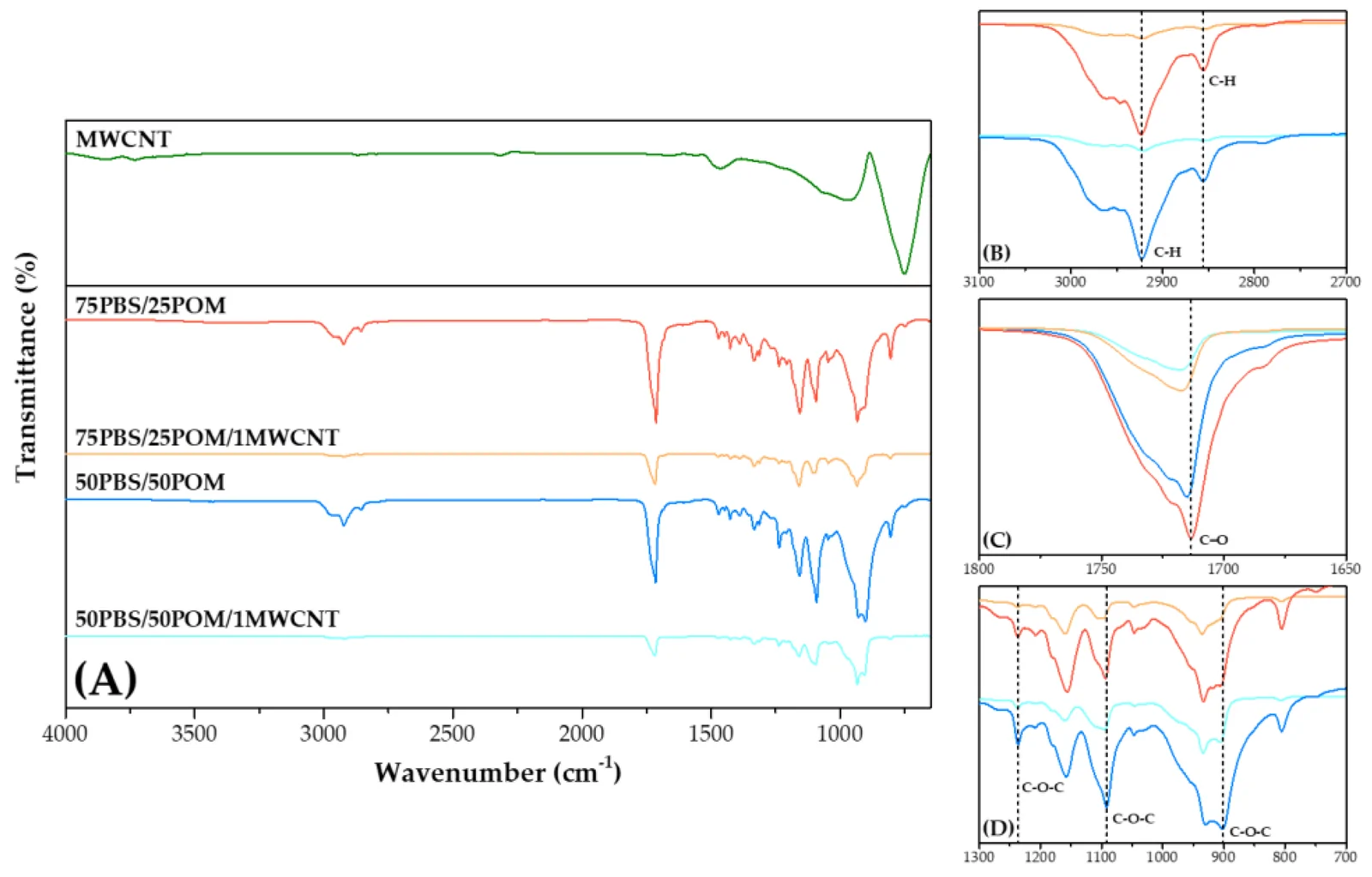
(**A**) FTIR spectra of MWCNT, PBS/POM and PBS/POM/1MWCNT nanocomposites; (**B**) 3100–2700 cm^−1^ region; (**C**) 1800–1650 cm^−1^ region; (**D**) 1300–700 cm^−1^ region.

In addition, the carbonyl (-C=O) stretching band of PBS at 1712 cm^−1^ shifted slightly to 1716 cm^−1^ upon the incorporation of 1 wt% MWCNTs (Figure 2C). This shift may reflect a change in the local chemical environment of PBS carbonyl groups resulting from their proximity to the MWCNT surface and may be indicative of physical interactions between PBS and MWCNTs [44,45]. Nevertheless, considering the small magnitude of the shift, this observation alone cannot be regarded as definitive evidence of a specific intermolecular interaction. Likewise, in POM/MWCNT systems, the C–O–C stretching bands originally observed at 1236, 1088, and 894 cm^−1^ exhibited slight shifts toward higher wavenumbers, which may be associated with changes in the local molecular environment of POM chains near the MWCNT surfaces (Figure 2D). Similar small peak shifts were also observed upon the incorporation of MWCNTs into PBS/POM blends. The FTIR spectra of the PBS/POM/MWCNT nanocomposites are presented in Figure 3. As a general conclusion, small shifts (~1–2 cm^−1^) in the presence of MWCNTs reflect physical entrapment and distribution within the polymer matrix rather than definitive chemical bonding.

### 3.2. Scanning Electron Microscopy (SEM)

**Figure 4:**
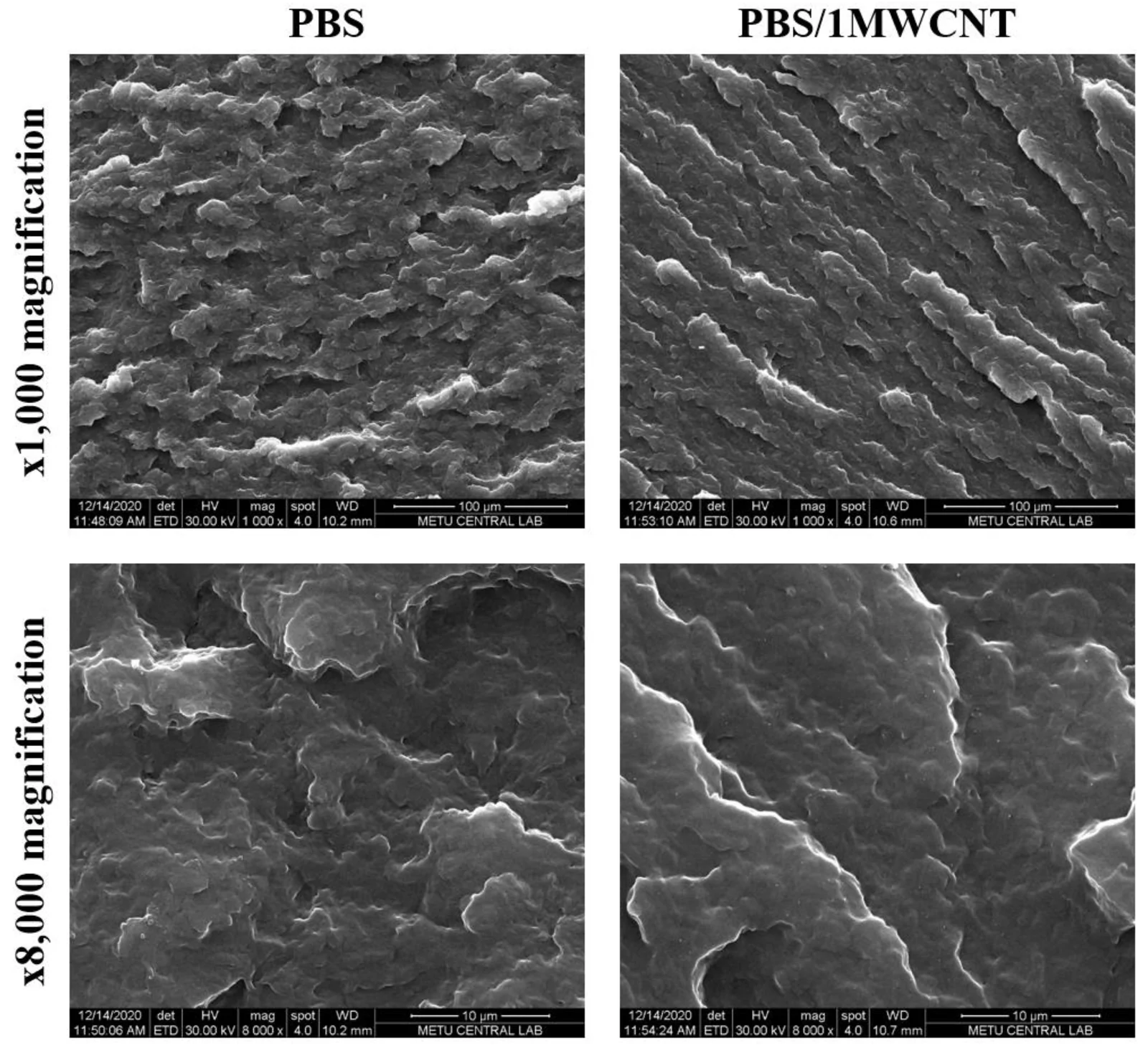
Cryogenically fractured surface morphologies of PBS and PBS/1MWCNT nanocomposites.

**Figure 5:**
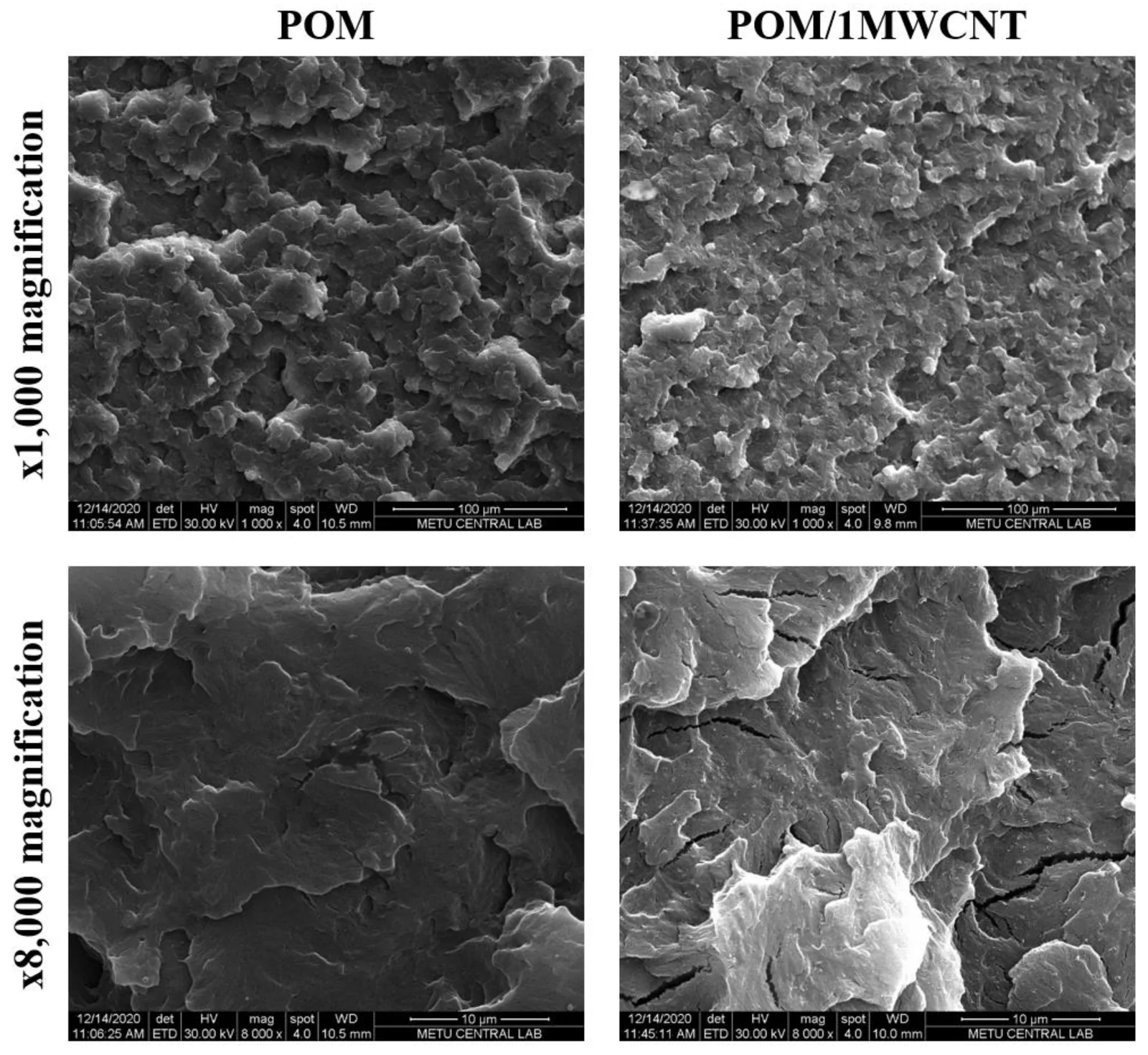
Cryogenically fractured surface morphologies of POM and POM/1MWCNT nanocomposites.

Scanning electron microscopy (SEM) was used to examine the surface morphology of the PBS/POM blends and to qualitatively assess the distribution of MWCNTs within the polymer matrices. In the SEM images of neat PBS (Figure 4), a relatively smooth and continuous surface morphology was observed. Following the incorporation of MWCNTs, the PBS/MWCNT nanocomposites generally retained a similar surface morphology, and no large MWCNT agglomerates were readily apparent at the length scale resolved by SEM. However, given the spatial resolution of SEM and the absence of complementary TEM observations, the SEM images do not allow the MWCNTs to be described as definitively or uniformly dispersed at the nanoscale. Similarly, the POM/MWCNT nanocomposites (Figure 5) exhibited a relatively continuous surface morphology, with no pronounced micron-scale agglomerates readily discernible in the examined regions. The presence of localized fibrillar structures in the SEM images of neat POM is indicative of plastic deformation, while the neat POM generally exhibited a relatively smooth morphology similar to that of PBS. Upon MWCNT incorporation, the surface morphology appeared somewhat more brittle.

**Figure 6:**
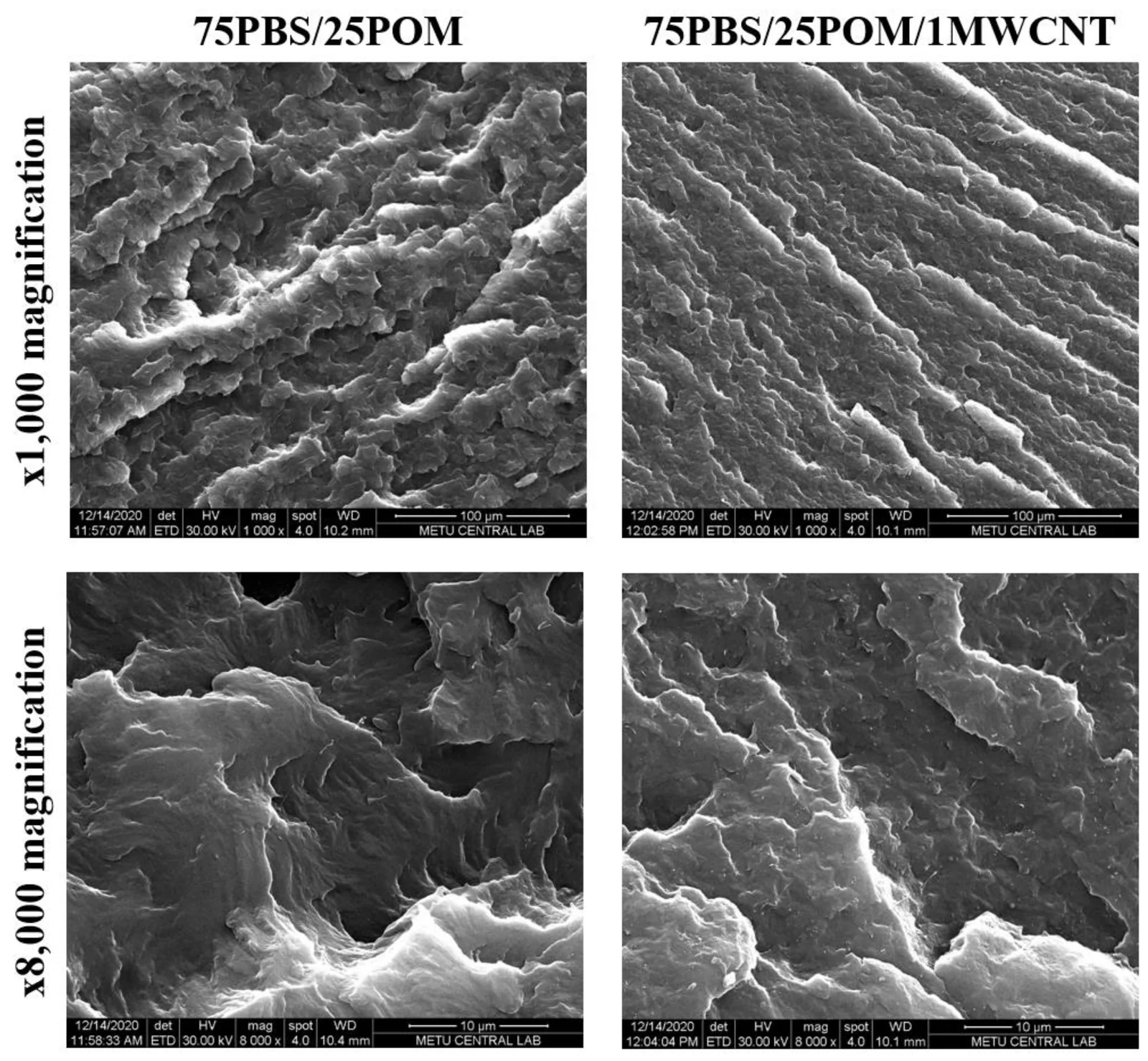
Cryogenically fractured surface morphologies of 75PBS/25POM and 75PBS/25POM/1MWCNT nanocomposites.

**Figure 7:**
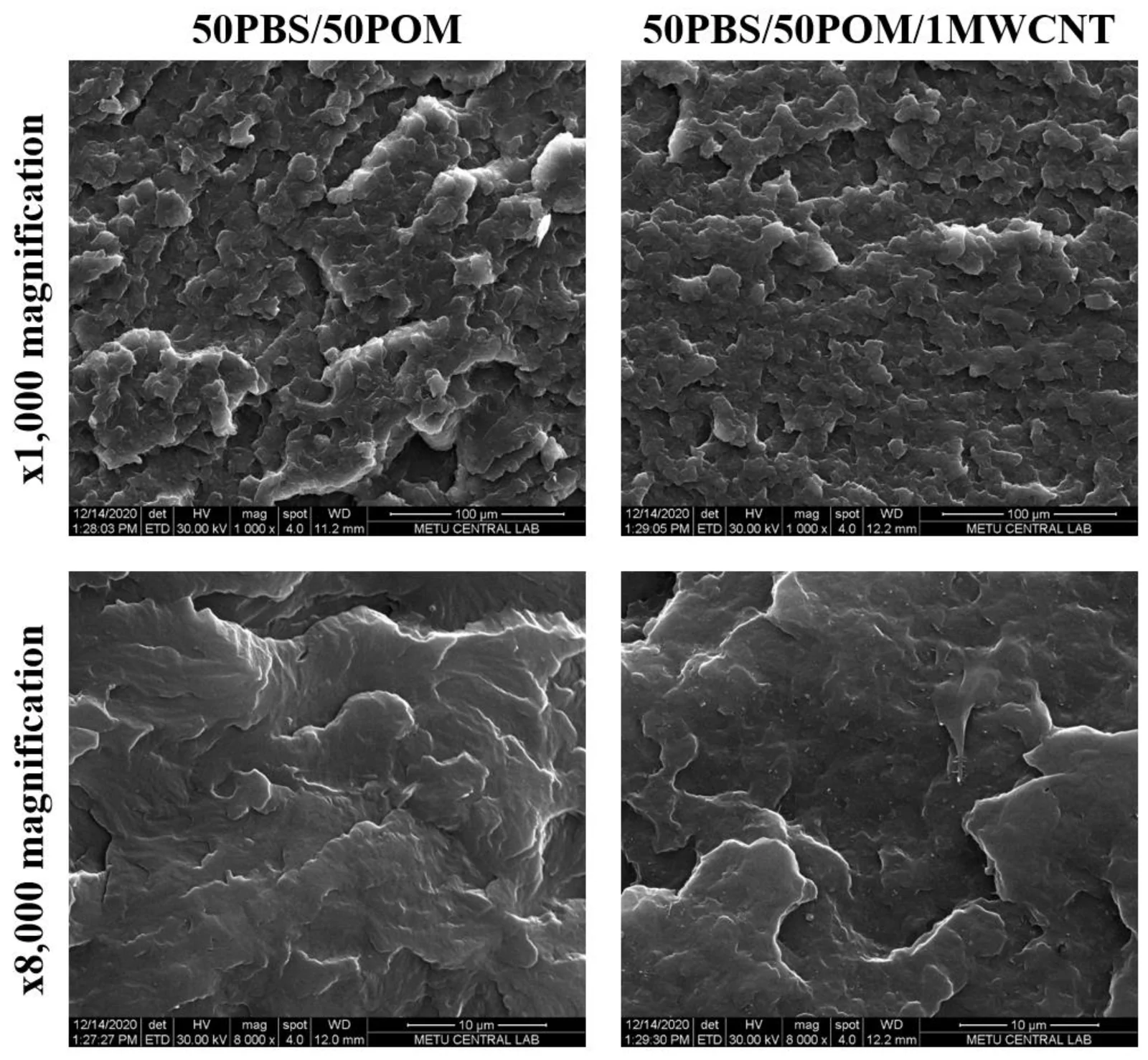
Cryogenically fractured surface morphologies of 50PBS/50POM and 50PBS/50POM/1MWCNT nanocomposites.

As shown in the SEM images of the PBS/POM blends (Figure 6 and Figure 7), no obvious micron-scale phase separation, large interfacial voids, or pronounced debonding was observed, irrespective of the blend composition. Instead, the blends exhibited a relatively continuous morphology at the length scale accessible by SEM, suggesting reasonably good interfacial continuity between the PBS and POM. The surface morphology became progressively smoother with increasing POM content, reflecting composition-dependent changes in the blend microstructure. These morphological observations are broadly consistent with the FTIR results, which suggest changes in the local molecular environment and possible physical interactions between the two polymers.

The localization of nanoparticles within the components of a polymer blend is one of the factors influencing thermodynamic compatibility. The first step in predicting the localization of nanoparticles within an immiscible or partially miscible polymer blend is to evaluate their thermodynamic affinity toward each constituent phase [46]. The preferential localization of nanoparticles is commonly assessed using the wetting parameter ($\omega_{a}$), which provides a quantitative measure of their thermodynamic preference for the blend components. The wetting parameter is determined according to Young’s equation as follows [33,34]:

(2) $$\omega_{a}=\frac{\gamma_{S-A}-\gamma_{S-B}}{\gamma_{A-B}}$$

where $\gamma_{S-A}$ and $\gamma_{S-B}$ represent the interfacial tensions between the nanoparticle and polymers A and B, respectively. The localization of nanoparticles within the polymer blend can be predicted based on the value of the ($\omega_{a}$). Specifically, when $\omega_{a}$ > 1, the nanoparticles are thermodynamically favored to localize within polymer A, whereas $\omega_{a}$ < −1 indicates preferential localization within polymer B. For −1 < $\omega_{a}$ < 1, the nanoparticles are expected to preferentially reside at the interface between polymers A and B, thereby minimizing the overall interfacial free energy of the blend. The interfacial tension between the blend components was determined by first calculating the surface free energies and their corresponding dispersive (Lifshitz–Van der Waals, LW) and polar (acid–base, AB) components using Equation (3):

(3) $$\gamma_{1-2}=\gamma_{1}+\gamma_{2}-2\left[\sqrt{\left(\gamma_{1}^{LW}\gamma_{2}^{LW}\right)}+\sqrt{\left(\gamma_{1}^{AB}\gamma_{2}^{AB}\right)}\right]$$

The surface energy values of PBS and POM were obtained by calculating the contributions of basic ($\gamma^{-}$), acidic ($\gamma^{+}$), polar $\left(\gamma^{AB}\right)$ ve dispersive $\left(\gamma^{LW}\right)$ components, as well as the contact angle (θ) data, using Equation (4):

(4) $$\left(1+{\cos\theta }_{L}\right)\gamma_{L}^{TOT}=2\left[{\left(\gamma_{L}^{LW}\gamma_{S}^{LW}\right)}^{1/2}+{\left(\gamma_{L}^{+}\gamma_{S}^{-}\right)}^{1/2}+{\left(\gamma_{L}^{-}\gamma_{S}^{+}\right)}^{1/2}\right]$$

The contact angles of the samples used to determine the surface energy values of the components are given in Table 2. The surface energies of PBS, and POM were then estimated by using Equation (3) and Equation (4), respectively and the results are reported in Table 3. The surface energy components of the MWCNT were obtained from the literature [35].

Table 4 shows the interfacial energies between the polymer–MWCNT, PBS-POM and the wetting parameter. As revealed by the selective localization analysis, the calculated wetting parameter ($\omega_{a}$) falls within the range of −1 < $\omega_{a}$ < 1, indicating the preferential localization of MWCNTs at the PBS/POM interface. The interfacial localization of MWCNTs is expected to inhibit the coalescence of the dispersed phase of POM and reduce the interfacial tension between the two polymers, thereby enhancing interfacial stability. As a result, the mechanical and thermal properties of the blends are significantly affected.

However, the absence of obvious micron-scale phase separation in the SEM images should not be interpreted as evidence of complete miscibility between PBS and POM. In particular, the distinct crystallization peaks observed for the PBS and POM phases in the DSC analysis indicate that the two polymers retain their individual crystalline phases, supporting the partial miscibility of the blend. Therefore, the SEM observations mainly indicate that no pronounced micron-scale phase separation is apparent under the investigated conditions, rather than demonstrating complete molecular-level miscibility. In addition, the wetting parameter analysis provides a thermodynamic indication of the preferential localization of MWCNTs at the PBS/POM interface. However, this analysis should be regarded as a predictive assessment rather than direct experimental evidence of MWCNT localization, since some of the surface-energy parameters used in the calculation were obtained from the literature and no direct microscopic measurement of nanotube localization was performed.

For the ternary PBS/POM/MWCNT systems, the SEM images (Figure 6 and Figure 7) exhibited a relatively continuous surface morphology, with no pronounced micron-scale MWCNT agglomerates readily discernible within the examined regions. These observations indicate a relatively uniform distribution of the MWCNT phase at the spatial scale accessible by SEM. The relatively continuous morphology observed in the SEM images may be conducive to more effective stress transfer between the polymer matrix and the MWCNT phase; however, such an effect cannot be directly inferred from the morphological observations alone and should be evaluated in conjunction with the corresponding mechanical properties.

### 3.3. Mechanical Properties

**Figure 8:**
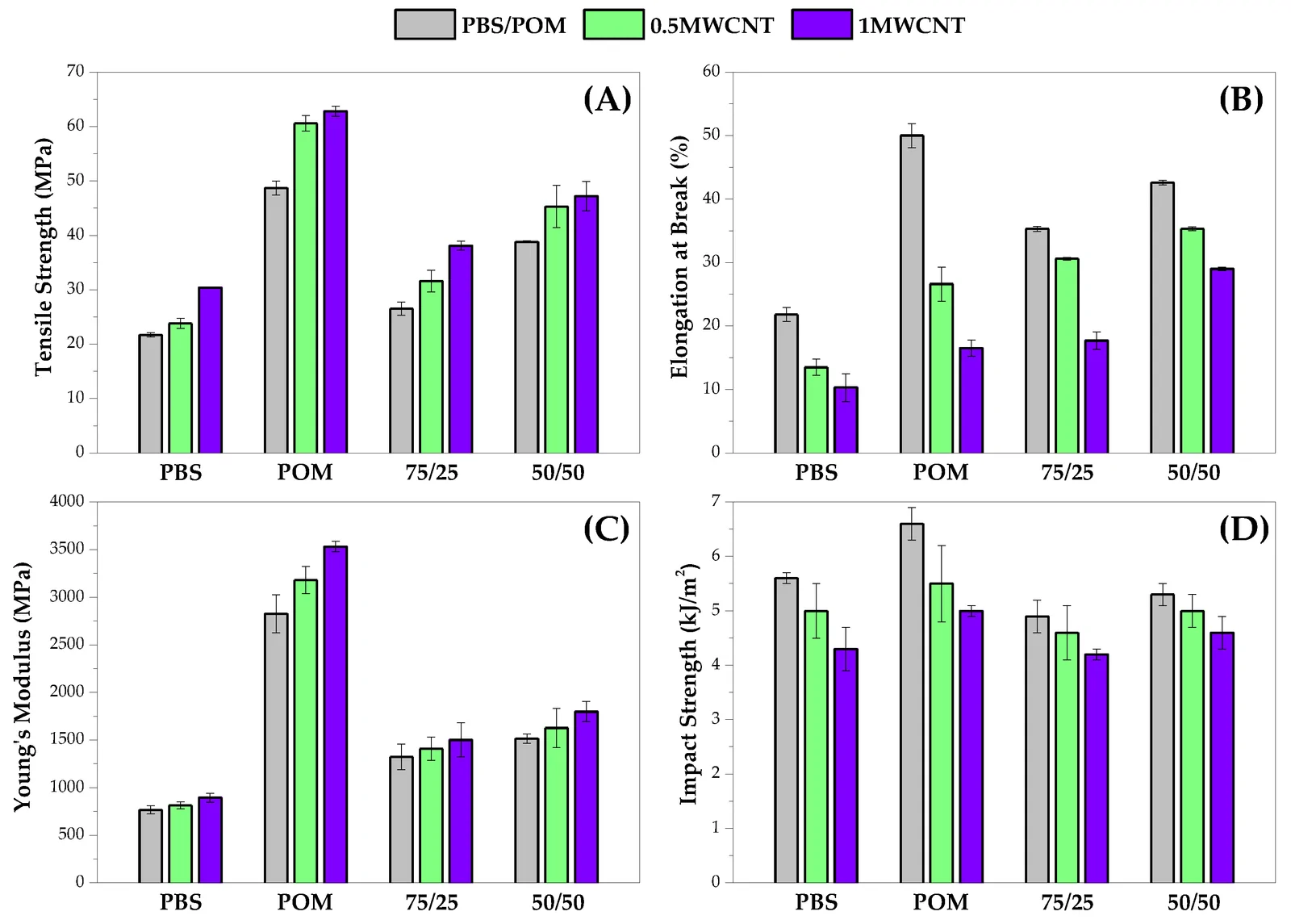
(**A**) Tensile strength. (**B**) Elongation at break. (**C**) Young’s modulus. (**D**) Impact strength of samples.

The mechanical properties of neat POM, neat PBS, PBS/POM blends with different composition ratios, and MWCNT-reinforced PBS, POM, and PBS/POM nanocomposites were characterized using tensile and Izod impact tests. The influence of blend composition and MWCNT incorporation on the mechanical behavior of the materials is presented in Figure 8, whereas the detailed mechanical test results are listed in Table 5.

The mechanical performance of polymer blends is strongly influenced by the morphology of the continuous phase, the size and distribution of the dispersed phase, and the interfacial interactions between the constituent phases. The tensile strength values of the PBS/POM blends were intermediate between those of the corresponding neat polymers, which can be attributed to the partial compatibility between PBS and POM. As shown in Figure 8A, the tensile strength of the PBS/POM blends increased progressively with increasing POM content, approaching the tensile strength of neat POM. This behavior can be attributed to the higher crystallinity and inherently superior mechanical properties of POM compared with PBS. The observed improvement in tensile strength may also be associated with the relatively continuous interfacial morphology of the blends and possible physical interactions between the PBS and POM phases. The FTIR results suggest changes in the local molecular environment upon blending, while the SEM observations revealed no pronounced micron-scale phase separation or interfacial discontinuities. The incorporation of MWCNTs into both neat PBS and neat POM resulted in a pronounced increase in tensile strength, which was directly proportional to the MWCNT loading. In particle-reinforced composites, tensile strength is governed primarily by the efficiency of stress transfer between the matrix and the reinforcing particles. Well-dispersed/distributed fillers with strong interfacial adhesion enable the effective transfer of the applied stress from the polymer matrix to the reinforcing phase, thereby enhancing the load-bearing capability and mechanical strength of the composite [47]. The SEM observations showed no pronounced micron-scale MWCNT agglomerates within the examined regions, even at a loading level of 1 wt.% MWCNT, suggesting a relatively favorable distribution of MWCNTs at the spatial scale accessible by SEM. The relatively favorable MWCNT distribution, together with possible physical interactions between the polymer chains and MWCNT surfaces, may have contributed to more effective stress transfer and, consequently, to the observed enhancement in tensile strength. The addition of MWCNT was found to have a more pronounced effect on the tensile strength of PBS. For example, the tensile strength of neat PBS increased by approximately 40% with the addition of 1 wt% MWCNT, whereas the tensile strength of neat POM increased by only 29%. The increases in the tensile strength of PBS observed at especially low MWCNT loading levels are consistent with the findings reported by Fathilah Binti Ali and Raja Mohan [48]. Consistent with the tensile behavior observed for the POM/MWCNT and PBS/MWCNT nanocomposites, the incorporation of MWCNTs into the PBS/POM blend resulted in a further enhancement in tensile strength. This improvement may be associated with the relatively favorable distribution of MWCNTs at the spatial scale accessible by SEM, which may contribute to more effective stress transfer within the blend.

The elongation at break values of the samples are presented in Figure 8B. The incorporation of POM into PBS led to an increase in elongation at break, indicating enhanced deformability of the blends. This increment in elongation at break values of PBS/POM blends as the POM content increased can be attributed to the physical interactions between PBS and POM. In contrast, a gradual reduction in elongation at break was observed for all samples with increasing MWCNT content (Table 5). This reduction can be attributed to the homogeneous distribution of MWCNTs and the increased polymer chain entanglement, which effectively restricts polymer chain mobility. In addition, the intrinsically rigid nature of MWCNTs contributes to increased stiffness and reduced ductility. Consequently, the presence of MWCNTs impedes chain rearrangement and deformation under tensile loading, resulting in lower elongation at break values [45,49].

In addition, as expected, the Young’s modulus of the PBS/POM blends increased with increasing POM content due to the higher crystallinity and greater chain entanglement density of POM, which enhance the stiffness of the blends (Figure 8C). When the modulus values of neat POM, neat PBS, and PBS/POM blends containing MWCNTs were compared with those of their unreinforced counterparts, significantly higher values were obtained in the presence of MWCNTs. This improvement can be attributed to two main factors. First, according to composite mixture rules, the incorporation of high-modulus particulate fillers leads to an increase in the overall stiffness of the resulting material, as the modulus of the reinforcing phase is considerably higher than that of the polymer matrix. Second, the non-covalent interactions between the matrix and the reinforcing phase play a crucial role in determining the mechanical performance of polymer nanocomposites. A homogeneous distribution of nanoparticles within the polymer matrix creates a larger interfacial area between the reinforcing agent and the polymer matrix, thereby enhancing stress transfer efficiency between the phases and leading to improved stiffness. Even at a relatively low MWCNT loading of 1 wt.%, the uniform distribution of carbon nanotubes within the POM, PBS, and PBS/POM matrices resulted in a pronounced increase in Young’s modulus due to the enlarged polymer–MWCNT interfacial area and improved load transfer efficiency.

The impact strength of polymer composites is influenced by several factors, including filler incorporation, filler–matrix interfacial interactions, and the degree of crystallinity. As shown in Figure 8D, the addition of POM to the PBS matrix led to a reduction in impact strength. This behavior can be attributed to changes in blend morphology and the increase in spherulite density associated with the presence of POM, which generally results in a more rigid and less impact-resistant structure. Furthermore, the incorporation of MWCNTs caused a general decrease in impact strength across all systems. This reduction can be associated with the inherently stiff nature of MWCNTs and their reinforcing effect, which increases the rigidity and the crystallinity of the polymer matrix. As a consequence, the ability of the composites to dissipate and absorb energy under sudden loading conditions is diminished, leading to lower impact resistance.

### 3.4. Dynamical Mechanical Analysis (DMA)

**Figure 9:**
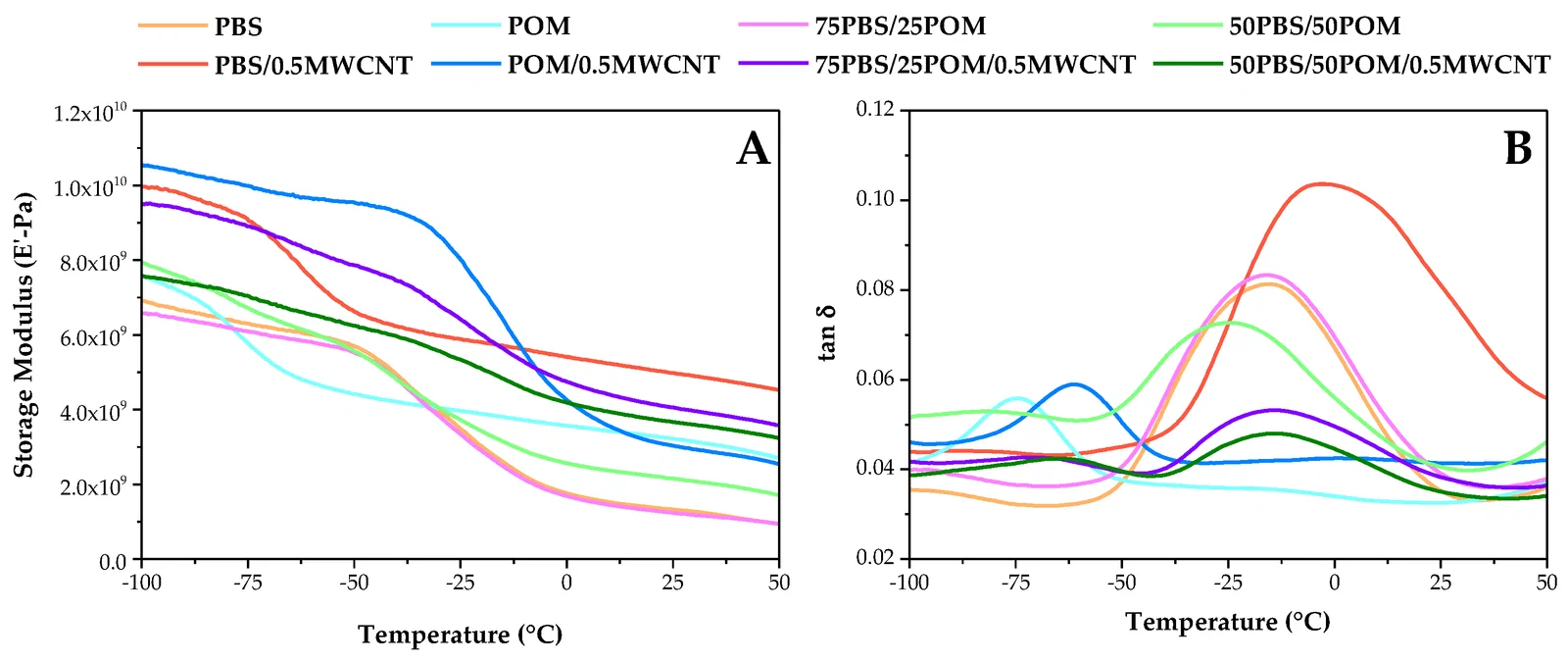
Changes in (**A**) storage modulus and (**B**) tan δ of the samples as a function of temperature.

Figure 9 illustrates the viscoelastic behavior of neat POM, neat PBS, and their binary blends formulated at various composition ratios, alongside the corresponding MWCNT-reinforced binary and ternary nanocomposites (PBS/MWCNT, POM/MWCNT, and PBS/POM/MWCNT, including 0.5 wt.%MWCNT). As expected, the addition of POM led to an enhancement in the storage modulus of the PBS/POM blends, an effect that is especially pronounced in the glassy region (Figure 9A). This upward trend is directly attributable to the higher crystallinity and greater structural stiffness of POM compared with PBS. The incorporation of MWCNTs into both neat polymers and PBS/POM blends further enhanced the storage modulus relative to the unreinforced systems. This improvement is likely due to the nucleating effect of MWCNTs on POM and/or PBS crystallization, combined with the intrinsic rigidity of the carbon nanotubes. In addition, the relatively favorable distribution of MWCNTs within the PBS/POM matrix, as observed at the spatial scale accessible by SEM, may increase the effective filler–matrix interfacial area and facilitate stress transfer, thereby contributing to the observed enhancement in storage modulus [32].

The glass transition temperature (T_g_), which is associated with the mobility of polymer chains in the amorphous phase, was determined from the peak values of the tan δ curves as a function of temperature obtained from dynamic mechanical analysis (DMA) [50]. The T_g_ of neat POM and neat PBS was determined to be −75 ± 1 °C and −12 ± 1 °C, respectively (Figure 9B). For the PBS/POM blends, a single glass transition temperature was observed for both composition ratios. The presence of a single T_g_ indicates enhanced compatibility between the blend components and suggests a reduced degree of phase separation within the blend system. As also supported by the FTIR results, this behavior is attributed to favorable physical interactions between PBS and POM, confirming the partial miscibility of the blend constituents. In addition, a reduction in the tan δ peak intensity was observed with increasing POM content, indicating a decrease in damping capacity. This trend is consistent with the inherently lower energy dissipation ability of POM and becomes more pronounced as its fraction in the blend increases. The incorporation of MWCNTs induced a shift in T_g_ toward higher temperatures for both neat polymers. This behavior can be attributed to the restricted mobility of polymer chains caused by the presence of MWCNTs, which hinders segmental motion during the glass transition and thus shifts the relaxation process to higher temperatures [45]. Ashida and Noguchi established a relationship between tan δ and interfacial adhesion, where lower tan δ values indicate stronger interfacial interactions between the polymer matrix and the reinforcing phase. As shown in Figure 9B, the incorporation of MWCNTs into the PBS/POM blends led to a decrease in tan δ values. This reduction suggests improved interfacial interactions between the MWCNTs and the polymer matrices, indicating favorable physical interactions at the filler–matrix interface and more efficient stress transfer within the nanocomposites. In addition, the presence of MWCNTs contributed to a reduction in the dispersed-phase size, resulting in a finer and more homogeneous phase morphology, which may have further restricted polymer chain mobility and contributed to the observed decrease in tan δ values. Furthermore, the observed decrease in the tan δ values is considered to be associated with the increase in the crystallinity of both PBS and POM in the presence of MWCNT, as also discussed based on the XRD analysis results.

### 3.5. Non-Isothermal Crystallization Kinetics

**Figure 10:**
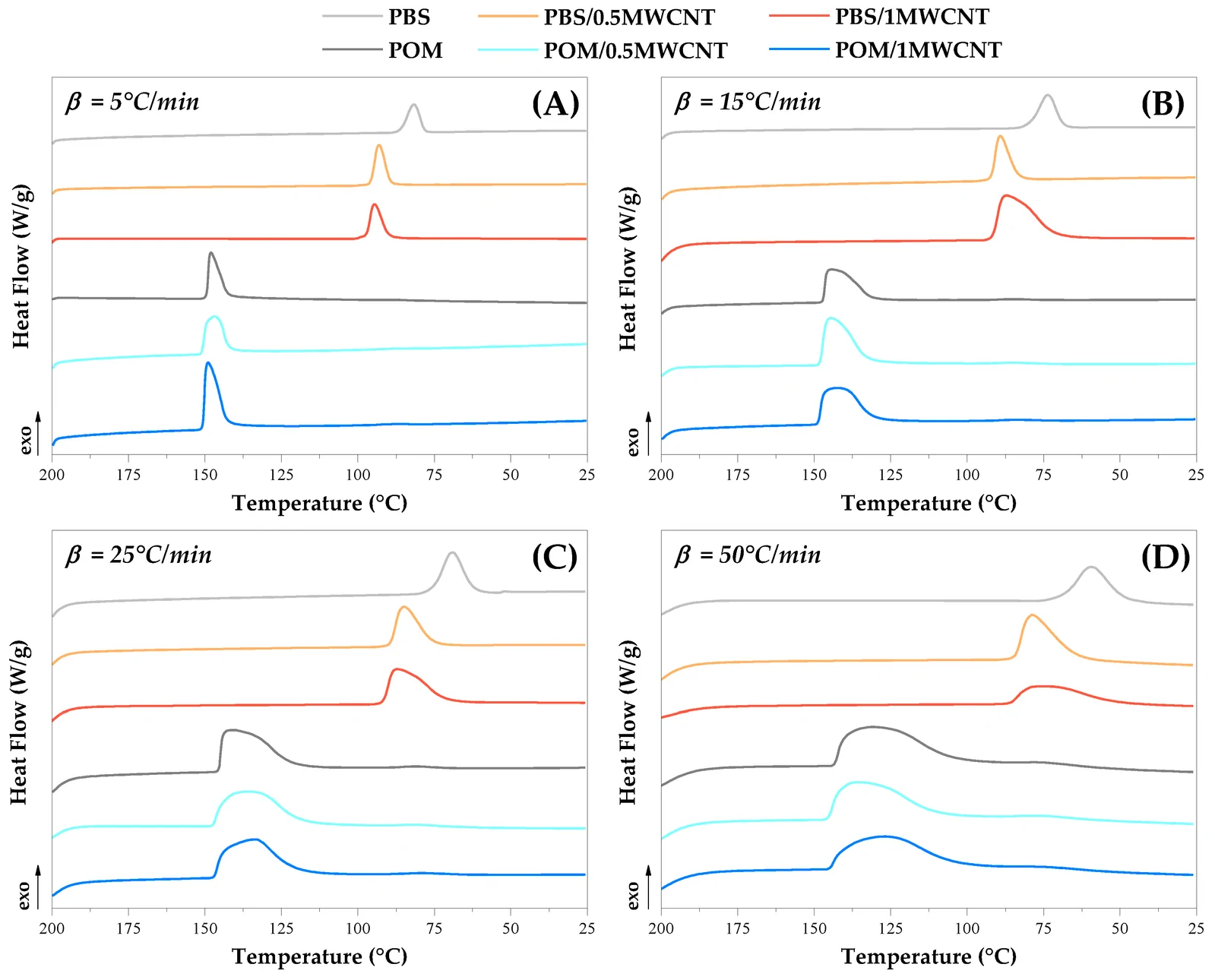
DSC cooling thermograms of PBS, POM, PBS/MWCNT and POM/MWCNT samples obtained at cooling rates of (**A**) 5 °C/min, (**B**) 15 °C/min, (**C**) 25 °C/min, and (**D**) 50 °C/min.

**Figure 11:**
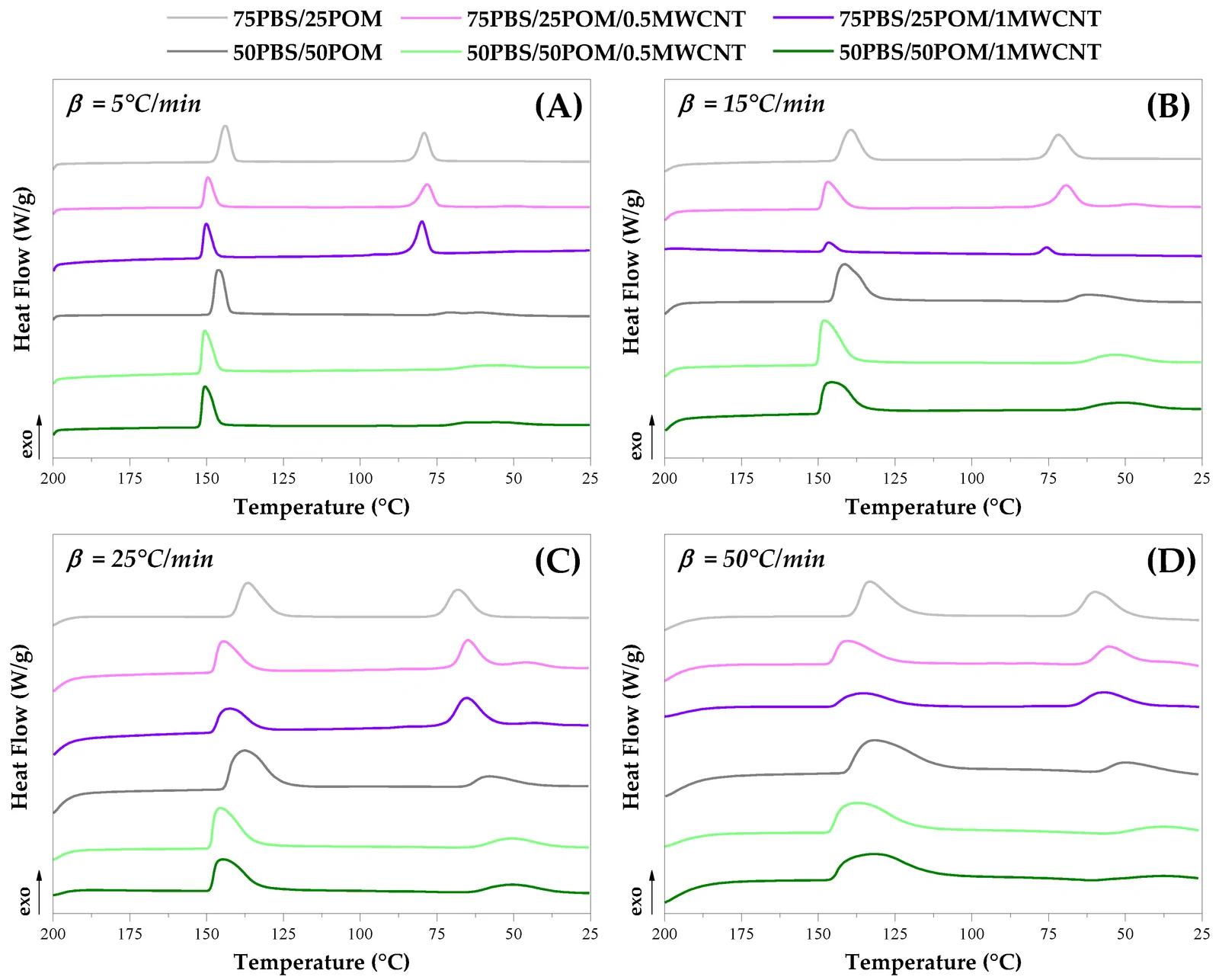
DSC cooling thermograms of PBS/POM and PBS/POM/MWCNT samples obtained at cooling rates of (**A**) 5 °C/min, (**B**) 15 °C/min, (**C**) 25 °C/min, and (**D**) 50 °C/min.

The non-isothermal melt crystallization DSC thermograms at different cooling rates (5, 15, 25, and 50 °C/min) for neat polymers, PBS/MWCNT, POM/MWCNT, and PBS/POM/MWCNT nanocomposites are presented in Figure 10 and Figure 11. The melt crystallization onset (T_c,onset_), melt crystallization peak (T_c,peak_), melt crystallization endset temperatures (T_c,endset_) and crystallization half-time (t_1/2_) values of neat PBS, neat POM, POM and PBS nanocomposites containing different amounts of MWCNT, and PBS/POM/MWCNT nanocomposites are listed in Table 6 and Table 7. The results obtained from the DSC analysis generally showed a standard deviation of approximately ±1 °C. As shown in Figure 10 and Figure 11 and Table 6 and Table 7, the melt crystallization onset temperatures of neat PBS and POM, as well as their blends and MWCNT-containing nanocomposites, shifted to lower temperatures with increasing cooling rates. Crystal formation and growth are time-dependent processes. When a polymer sample is cooled slowly from the melt, sufficient time is available for the nucleation and growth of crystalline embryos, enabling crystallization to initiate at relatively higher temperatures during the cooling process [51]. However, at high cooling rates, the mobility of polymer chains becomes insufficient to accommodate the rapid decrease in temperature [40]. Consequently, crystallization is initiated at lower temperatures during cooling and proceeds over a shorter time interval, resulting in more rapid completion of the crystallization process.

As shown in Figure 11, the DSC thermograms of the PBS/POM blends exhibited distinct crystallization peaks corresponding to each polymer component, indicating partial miscibility rather than complete miscibility between PBS and POM. Moreover, this behavior can also be attributed to the substantial difference between the melting temperatures of POM and PBS [21]. In the 75PBS/25POM blend, the crystallization onset temperatures of both POM and PBS were lower than those of the corresponding neat polymers, indicating that the presence of each polymer inhibited the crystallization of the other (Table 7). As expected, the T_c,onset_ values of both POM and PBS shifted to lower temperatures with increasing cooling rate. When the POM content was increased to 50%, the T_c,onset_ value of PBS decreased further, whereas that of POM approached its neat value. In summary, increasing the POM content significantly suppressed the crystallization of PBS because of the high crystallinity of POM, whereas the crystallization behavior of POM progressively approached that of the neat polymer. This finding indicates that PBS exhibits a nucleating effect on the crystallization of POM in the 50PBS/50POM blend.

In the 75PBS/25POM blend, the addition of 0.5 wt.% MWCNT increased the crystallization onset temperature of POM and acted as a nucleating agent; however, this effect disappeared at 1 wt.% MWCNT. This behavior can be attributed to the relatively reduced mobility of the POM chains with increasing MWCNT content. Nevertheless, compared with the 75PBS/25POM blend without MWCNT, the higher melt-crystallization onset temperature of POM still indicates that the presence of MWCNTs accelerates the crystallization of POM. However, in the 75PBS/25POM blend, the shift of the melt crystallization temperature of the PBS phase toward lower temperatures, particularly at higher cooling rates, indicates a decrease in the crystallization rate. This behavior is attributed to the fact that PBS remains in the molten state at the temperature at which POM begins to crystallize, while the presence of MWCNTs promotes the nucleation of POM. Similar crystallization behavior was observed in 50PBS/50POM blends. In the blends of 50PBS/50POM, incorporating 0.5% MWCNT led to a pronounced reduction in the crystallization temperature of PBS, while the T_c,onset_ value for POM increased. At a given cooling rate, the incorporation of 1 wt.% MWCNT did not result in any significant change in the crystallization temperatures of either PBS or POM. The most significant observation for the PBS/POM blends was that the preferential localization of MWCNTs at the PBS/POM interface enhanced the crystallization rate of POM, particularly at lower MWCNT contents.

For both neat PBS and neat POM, the incorporation of MWCNTs resulted in increases in the T_c,onset_ values at all investigated cooling rates, with a further increase observed with increasing MWCNT content. These shifts toward higher crystallization temperatures indicate the effective heterogeneous nucleating ability of MWCNTs in nanocomposites [40,52]. Furthermore, at both loading levels, MWCNT shifted the melt crystallization temperature of PBS to higher values by approximately 10–13 °C at a given cooling rate (Table 6). In contrast, the corresponding increase for POM was limited to only 1–2 °C in the presence of MWCNT. These results indicate that MWCNT is a more effective nucleating agent for PBS than for POM.

**Figure 12:**
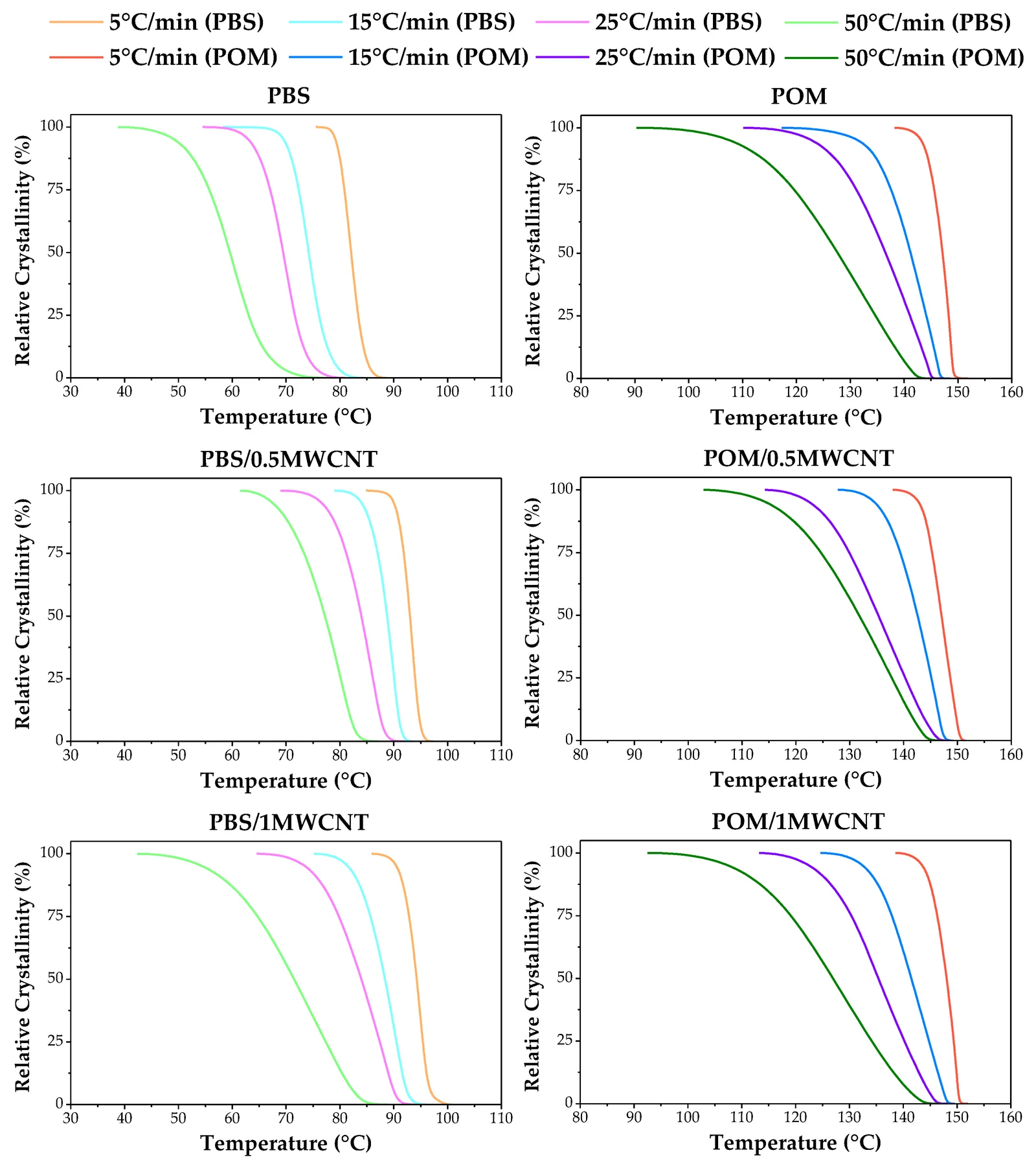
Changes in relative crystallinity, X(T), as a function of temperature at different cooling rates of PBS, POM, PBS/MWCNT and POM/MWCNT samples.

**Figure 13:**
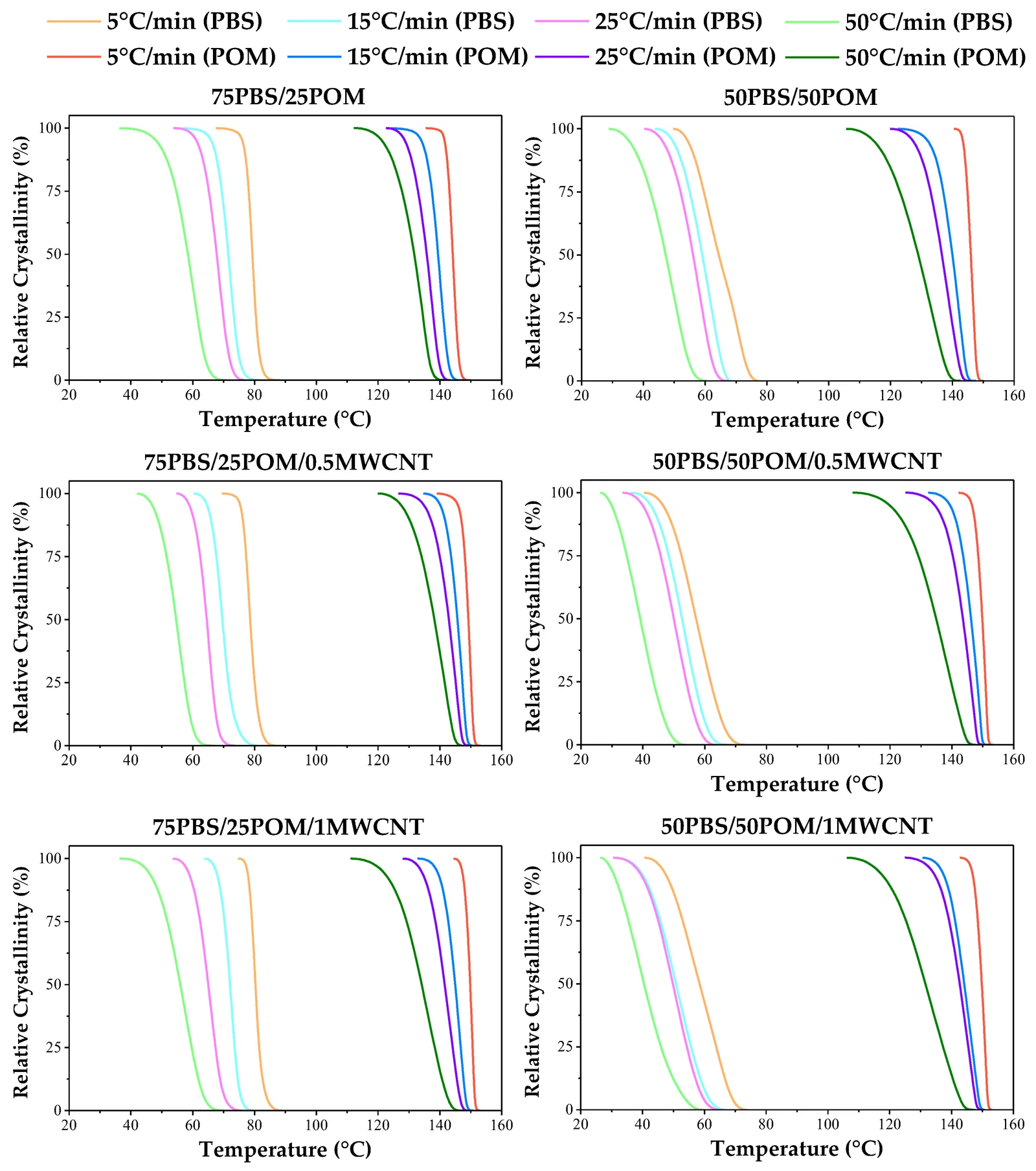
Changes in relative crystallinity, X(T), as a function of temperature at different cooling rates of PBS/POM and PBS/POM/MWCNT samples.

The relative crystallinity values can be calculated as a function of temperature or time. The relative crystallinity as a function of the crystallization temperature is defined by Equation (5):

(5) $$X\left(T\right)=\int_{T_{0}}^{T}\left(\frac{dH_{c}}{\mathrm{dT}}\right)\mathrm{dT}/\int_{T_{0}}^{T_{\infty }}\left(\frac{dH_{c}}{\mathrm{dT}}\right)\cdot \;\mathrm{dT}$$

where *T* represents the temperature during the crystallization process, *T*_0_ and *T_∞_* denote the crystallization onset and end temperatures, respectively, and *dHc* is the differential crystallization enthalpy. The relative crystallinity–temperature curves for neat PBS, neat POM, PBS/POM blends and PBS/POM nanocomposites (Figure 12 and Figure 13) exhibited a similar sigmoidal trend, indicating the thermal lag effect of cooling rate on crystallization. In other words, melt crystallization occurs at higher temperatures at lower cooling rates, whereas it occurs at lower temperatures at higher cooling rates. In general, at lower cooling rates, there is sufficient time for nucleation to be activated at higher temperatures [53]. The initial plateau corresponds to the nucleation stage, the linear region represents crystal growth, and the second plateau corresponds to secondary crystallization [54].

**Figure 14:**
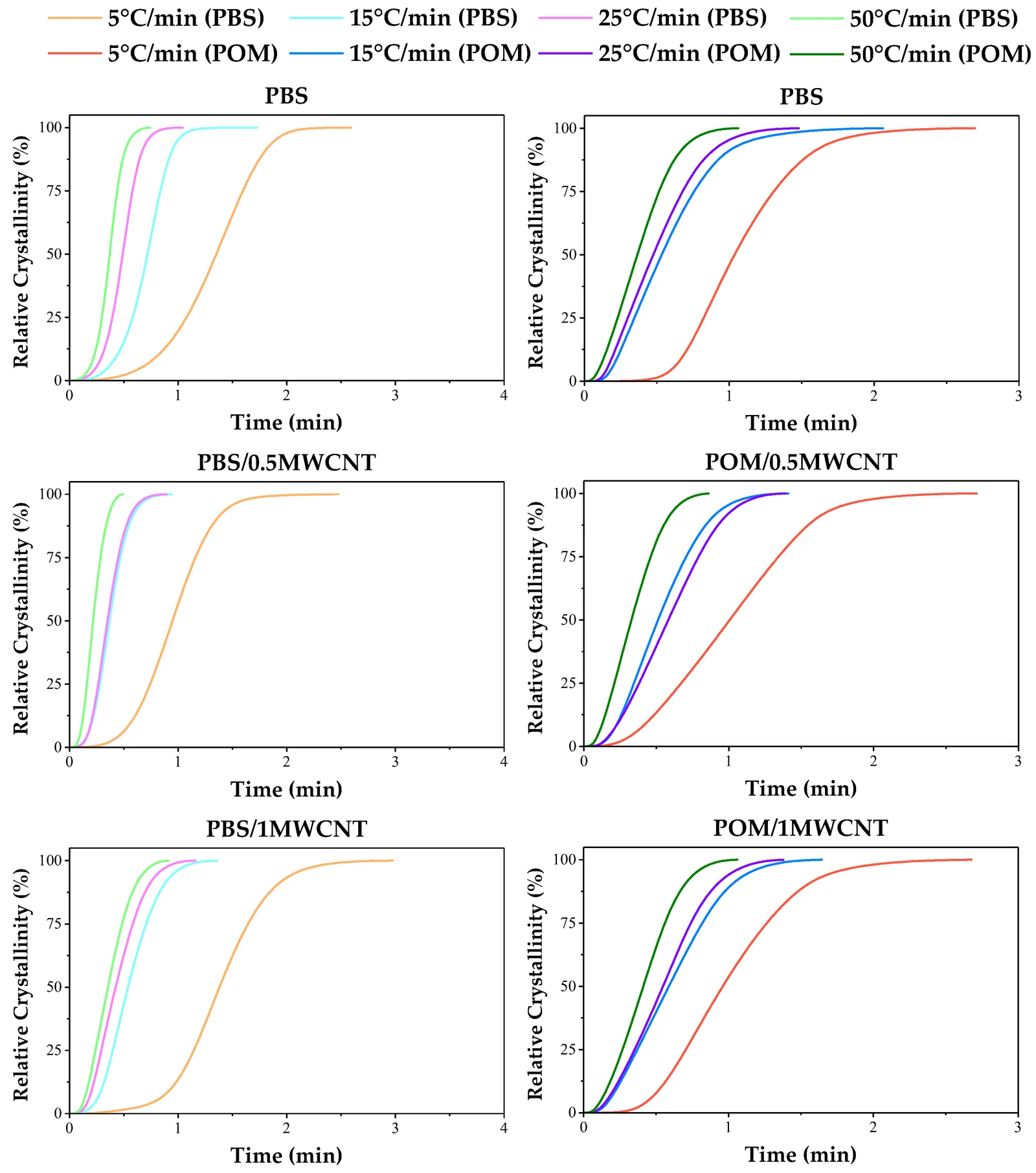
Changes in relative crystallinity, X(t), as a function of time at different cooling rates of PBS, POM, PBS/MWCNT and POM/MWCNT samples.

**Figure 15:**
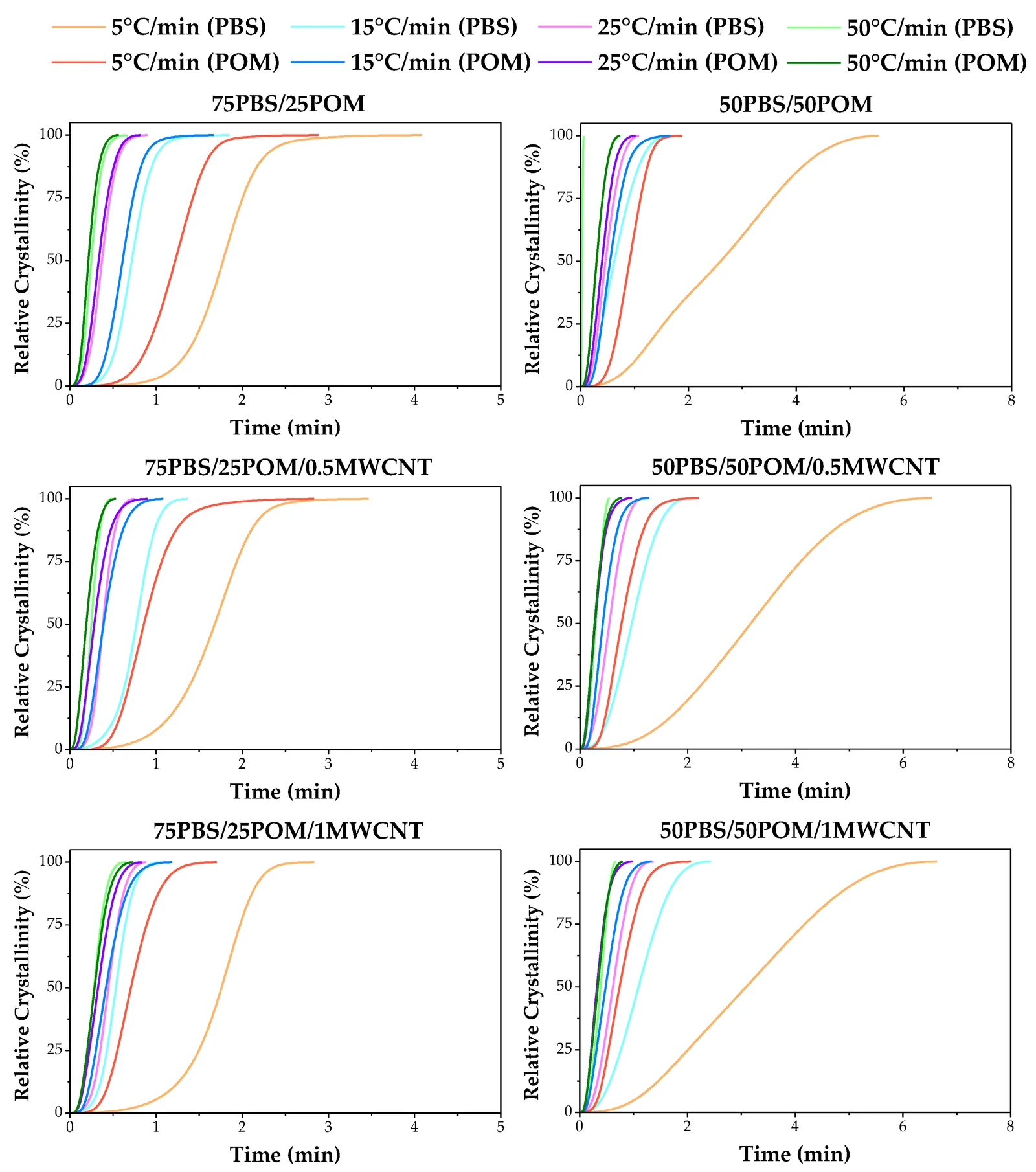
Changes in relative crystallinity, X(t), as a function of time at different cooling rates of PBS/POM and PBS/POM/MWCNT samples.

The crystallization time (*t*) for melt crystallization process of samples was calculated using Equation (6):

(6) $$t=\left(T_{0}-T\right)/\beta$$

where *T* is the temperature at the crystallization time *t*, and *β* is the cooling rate. This equation was used to determine the relationship between the relative crystallinity X(t) and crystallization time (t), as well as the crystallization half-time (t_1/2_). The relative crystallinity–time curves (Figure 14 and Figure 15) show that the curves become narrower and shift to the left with increasing cooling rates, whereas the slope of the linear region representing crystal growth increases. With increasing cooling rate, the crystallization half-time (t_1/2_), the time required to complete 50% of the crystallization, decreased, as crystallization was completed in a shorter time. For instance, increasing the cooling rate from 5 to 50 °C/min reduced the t_1/2_ value of neat PBS from 1.35 to 0.37 as seen in Table 6.

In PBS nanocomposites containing MWCNT, shorter crystallization half-times were generally obtained at a given cooling rate compared to neat PBS. These findings indicate that the presence of MWCNT enhances the crystallization rate of PBS. However, it was observed that increasing the MWCNT content from 0.5 to 1 wt.% resulted in an increase in the crystallization half-time. Nevertheless, the crystallization half-time remained lower than that of neat PBS, indicating that the presence of MWCNTs still promoted the overall crystallization kinetics of PBS. These results suggest that MWCNTs accelerate the crystallization rate of the PBS phase, especially at low concentrations. In POM/MWCNT nanocomposites, the increase in crystallization half-time observed regardless of MWCNT concentration is attributed to the higher number of spherulites formed in the presence of MWCNTs. This phenomenon is discussed in the polarized optical microscopy (POM) results. These findings show that MWCNT acts as a heterogeneous nucleating agent for PBS and POM [40,52].

For PBS/POM blends containing 25 wt% POM, an increase in the cooling rate resulted in shorter crystallization half-times, primarily because PBS and POM mutually suppressed each other’s crystallization. This mutual inhibition became more pronounced at higher cooling rates, leading to a reduction in the crystallization half-time of both phases. When the POM content was increased to 50 wt%, no clear trend was observed in the crystallization half-time of the PBS phase at a given cooling rate. In contrast, the crystallization half-time of the POM phase decreased, particularly at low cooling rates of 5 and 15 °C/min. This behavior can be attributed to the nucleating effect of the PBS phase on POM crystallization. In the presence of MWCNTs, lower crystallization half-times were generally observed for the POM phase, whereas higher crystallization half-times were obtained for the PBS phase. These results indicate that MWCNTs promote the crystallization of the POM phase while retarding the crystallization of the PBS phase.

**Figure 16:**
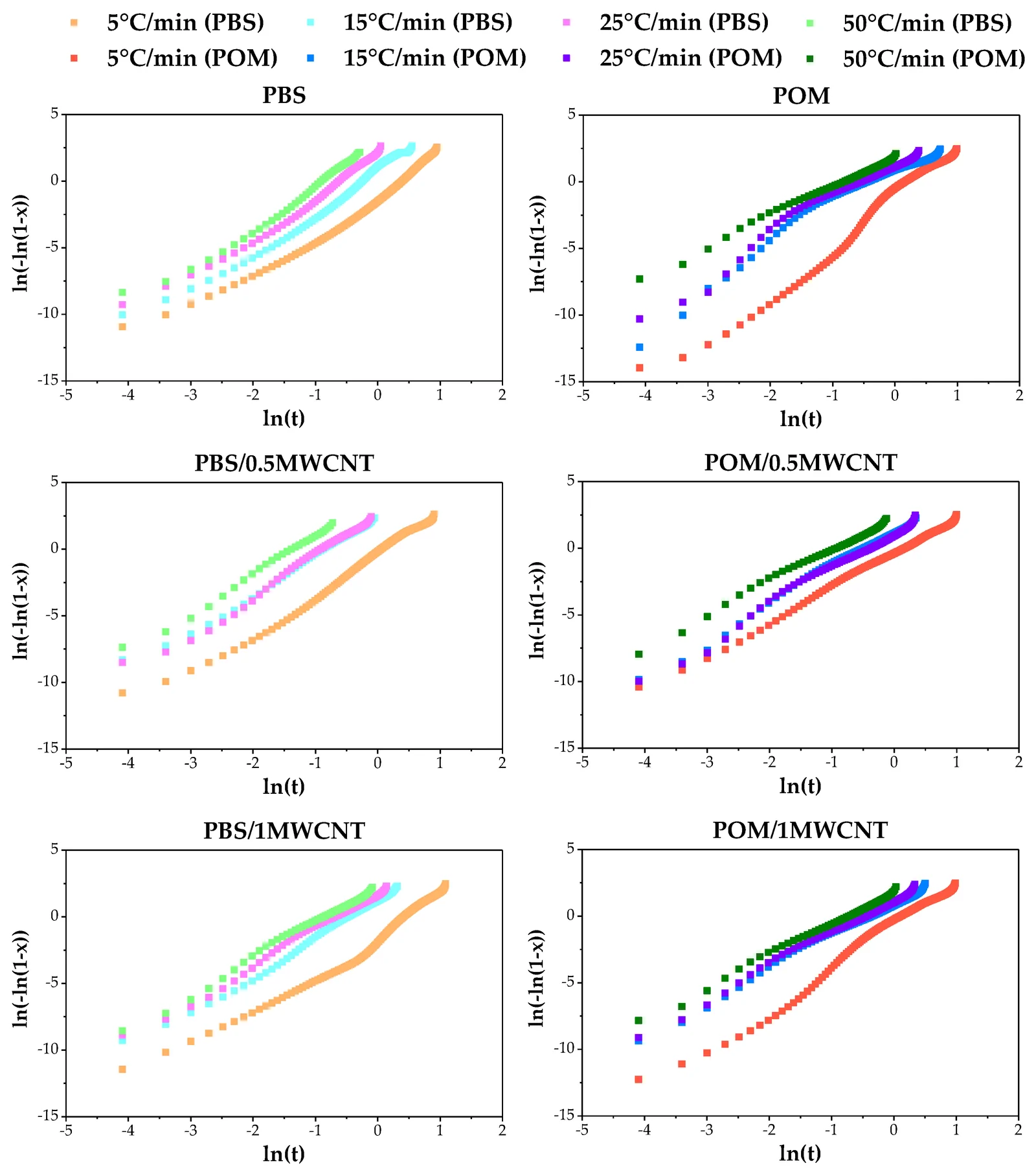
Avrami curves of the PBS, POM, PBS/MWCNT, and POM/MWCNT samples.

**Figure 17:**
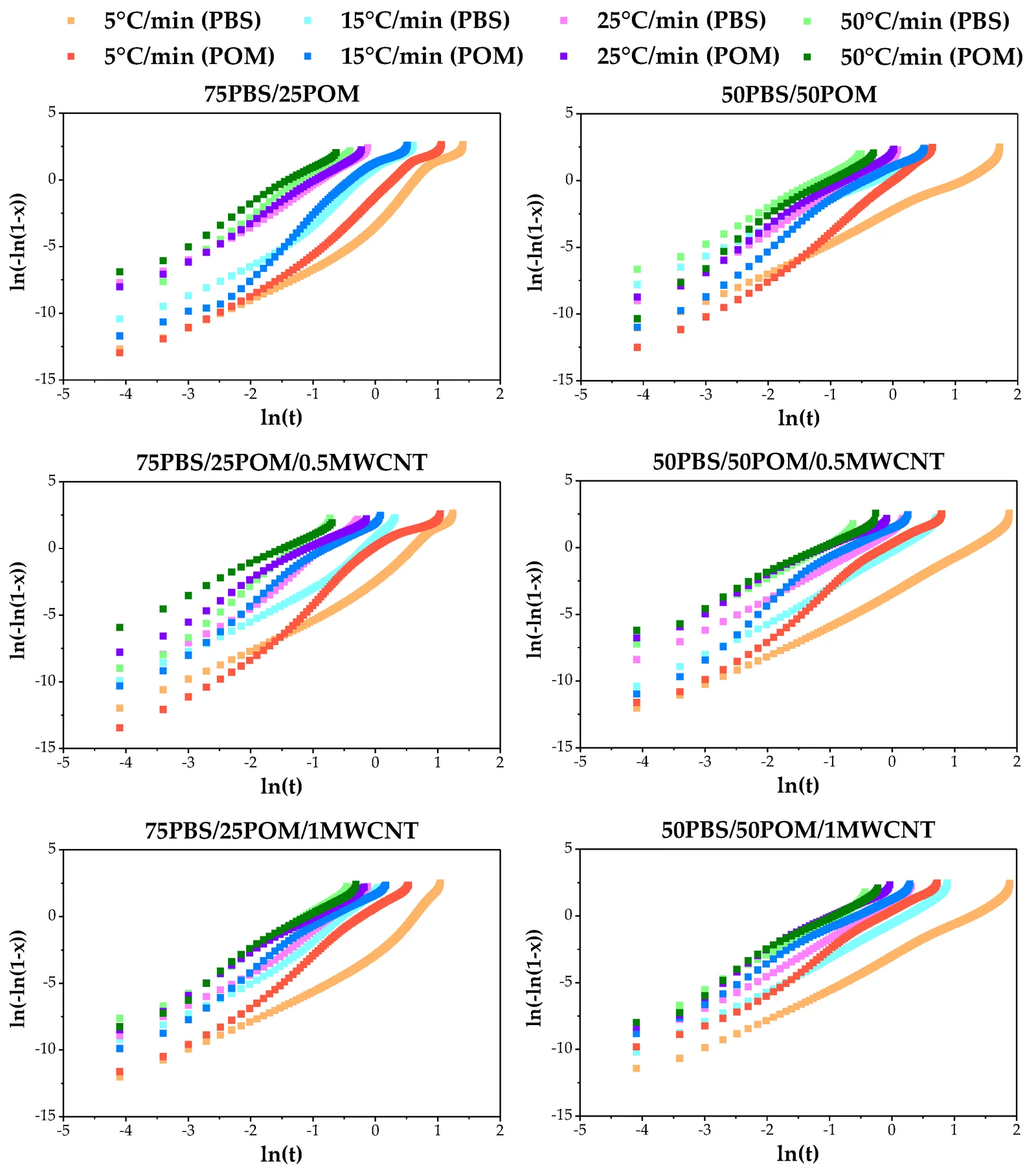
Avrami curves of the PBS/POM blends and PBS/POM/MWCNT nanocomposites.

The Avrami model is widely used to investigate the non-isothermal crystallization kinetics of semicrystalline polymers [55,56]. Non-isothermal crystallization consists of two stages, nucleation and crystal growth, both of which depend on temperature and cooling rate. The model is particularly applied to the linear region of the *X(t)–t* curve, where active crystal formation occurs (Figure 16 and Figure 17). The Avrami equation is expressed as follows (Equation (7)):

(7) $$1-X(t)=exp(-Zt^{n})$$

where *X(t)* represents the relative crystallinity, *Z* is the crystallization rate constant, and *n* is the Avrami exponent, which is related to the nucleation mechanism and crystal growth morphology. By applying a double logarithmic transformation, the following expression was obtained (Equation (8)):

(8) ln(−ln(1−X(t)))=lnZ+n⋅lnt

The values of *n* and *Z* were determined from the slope and intercept, respectively, of the plot of *In(−In(1 − X(t))* versus *ln t*. The Avrami plots for neat PBS, neat POM, and PBS or POM nanocomposites containing 0.5% and 1% MWCNT are presented in Figure 16, and those for the PBS/POM blends and PBS/POM/MWCNT nanocomposites with different compositions are shown in Figure 17. Crystallization in polymers occurs in two stages: primary and secondary crystallization, which appear as two distinct slopes on the Avrami plots. The primary crystallization stage corresponds to the onset of nucleation and nucleus growth. The secondary crystallization stage represents the transformation of the formed crystals into more perfect structures and is observed as a deviation from linearity in the Avrami plots. This deviation is attributed to the impingement of growing crystals and the resulting decrease in the crystallization rate.

According to the kinetic parameters presented in Table 8 and Table 9, the *n* values for neat POM, neat PBS and their nanocomposites including MWCNT range between 2 and 3, indicating that primary crystallization exhibits either two-dimensional (2D) or three-dimensional (3D) growth characteristics [40]. Similar findings were also observed for PBS/POM blends and PBS/POM/MWCNT nanocomposites.

However, it should be noted that the overall crystallization rate cannot be directly compared using the values of *n* and *Z* alone, since the Avrami exponent *n* is not constant (Table 8 and Table 9) and the unit of the crystallization rate constant depends on the value of *n*. Therefore, the crystallization half-time (t_0.5_), defined as the time required to reach 50% of the final crystallinity, is commonly used to evaluate and compare crystallization kinetics [57]. The crystallization half-time was calculated using Equation (9):

(9) $$t_{0.5}={\left(\frac{\ln2}{Z}\right)}^{1/n}$$

where *Z* is the crystallization rate constant and *n* is the Avrami exponent. The calculated values of *t*_0.5_ and its reciprocal (1/*t*_0.5_), which is often used as an indicator of the overall crystallization rate. As the cooling rate (β) decreased, *t*_0.5_ increased while 1/*t*_0.5_ decreased, indicating a reduction in the crystallization rate. This behavior was observed for both the neat polymer and the corresponding nanocomposite samples, suggesting that crystal formation becomes progressively slower at lower cooling rates.

The t_0.5_ and 1/t_0.5_ values calculated from the Avrami parameters were found to be in good agreement with the crystallization half-times previously obtained from the DSC thermograms and the relative crystallinity–time curves. For the PBS phase, the variations in t_0.5_ reproduced the trends observed in the non-isothermal crystallization analysis, where MWCNT incorporation generally resulted in shorter crystallization half-times compared to neat PBS, indicating enhanced crystallization kinetics. Likewise, the slight increase in t_0.5_ at higher MWCNT contents was observed. These findings indicate that MWCNTs act as heterogeneous nucleating agents for PBS. Surprisingly, the lower 1/t_0.5_ values observed for the POM/MWCNT nanocomposites suggest that MWCNT incorporation generally reduces the crystallization rate of POM. However, the POM analysis revealed that the spherulite density of the POM nanocomposite containing MWCNTs was considerably higher than that of neat POM. This seemingly contradictory trend in the 1/t_0.5_ values is attributed to the intrinsically high crystallization ability of neat POM.

Kinetic parameters obtained from the Avrami model confirmed that higher cooling rates reduced the crystallization half-time for both PBS and POM phases in blends with 25 wt.% POM. However, in the 50 wt.% POM blends, while the PBS phase displayed no discernible trend, the POM phase showed reduced t_0.5_ values, especially at lower cooling rates, indicating that PBS acts as a nucleating agent for POM crystallization. Additionally, the incorporation of MWCNTs decreased t_0.5_ (increased 1/*t*_0.5_) for the POM phase while increasing t_0.5_ (decreasing 1/*t*_0.5_) for the PBS phase. Overall, the nucleating influence of MWCNTs on the POM phase effectively hindered the crystallization of the PBS phase within the blends.

Unlike the Avrami model, the Ozawa method is also widely used to investigate non-isothermal crystallization kinetics. In the Avrami approach, the effects of cooling rate and the temperature variation with time are neglected. However, non-isothermal crystallization is significantly influenced by the cooling rate. To account for this effect, Ozawa modified the Avrami equation by incorporating the cooling rate term, resulting in the following expression (Equation (10)):

(10) $$ln\left(-\;ln\left(1-X\left(t\right)\right)\right)=ln\;K\left(T\right)-m\cdot ln\;\beta$$

where *m* is the Ozawa exponent, and *K(T)* is the Ozawa crystallization rate function. From the plot of ln(−ln(1 − *X(t)*)) versus ln*β*, the values of *m* and *K(T)* can be determined. For the Ozawa model to be applicable, the resulting plots should exhibit a linear relationship [58,59]. When linearity is achieved, the value of *m* can be obtained from the slope of the fitted line, while *K(T)* can be calculated from the intercept.

**Figure 18:**
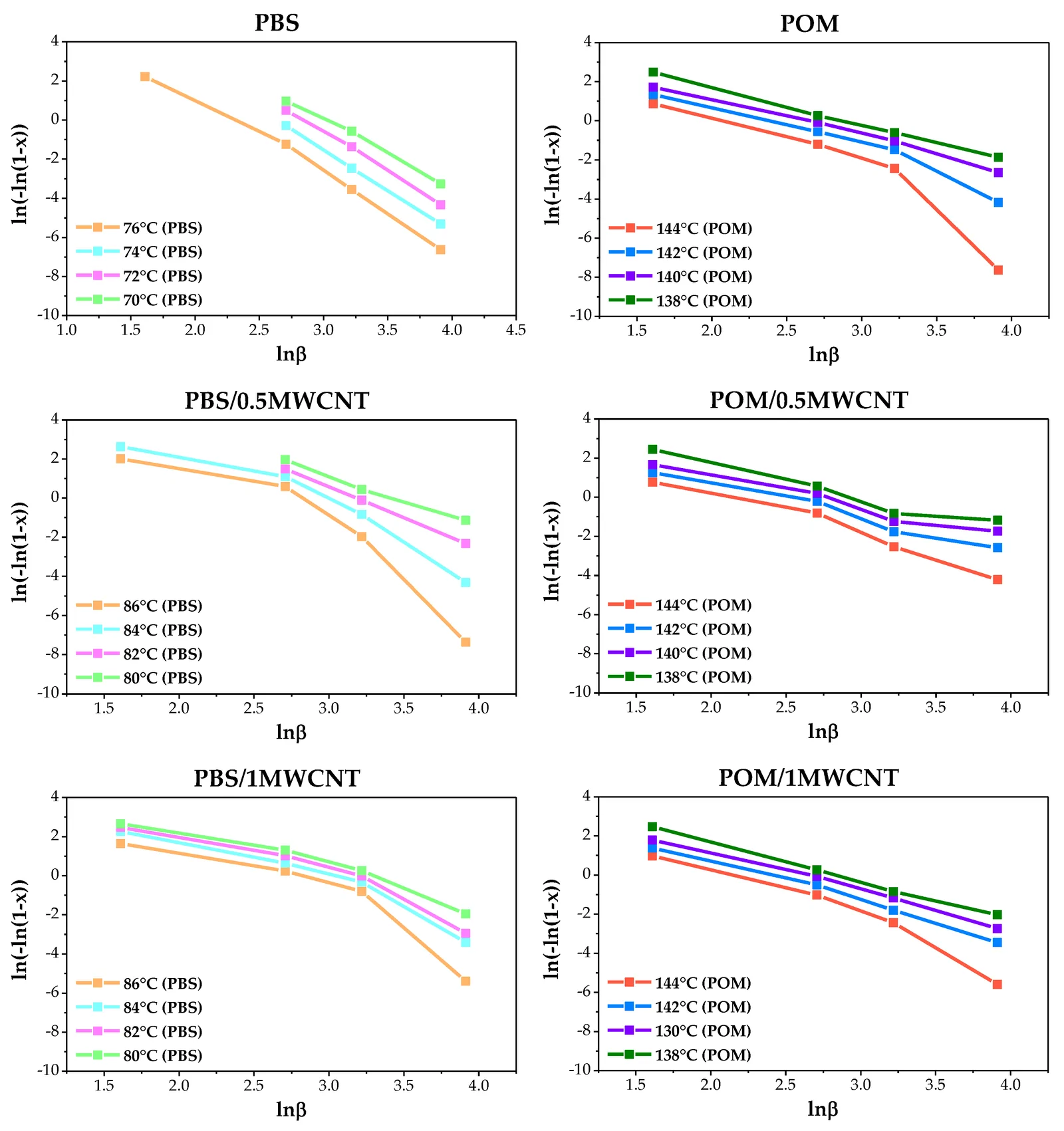
Ozawa curves of the PBS, POM, PBS/MWCNT, and POM/MWCNT samples.

**Figure 19:**
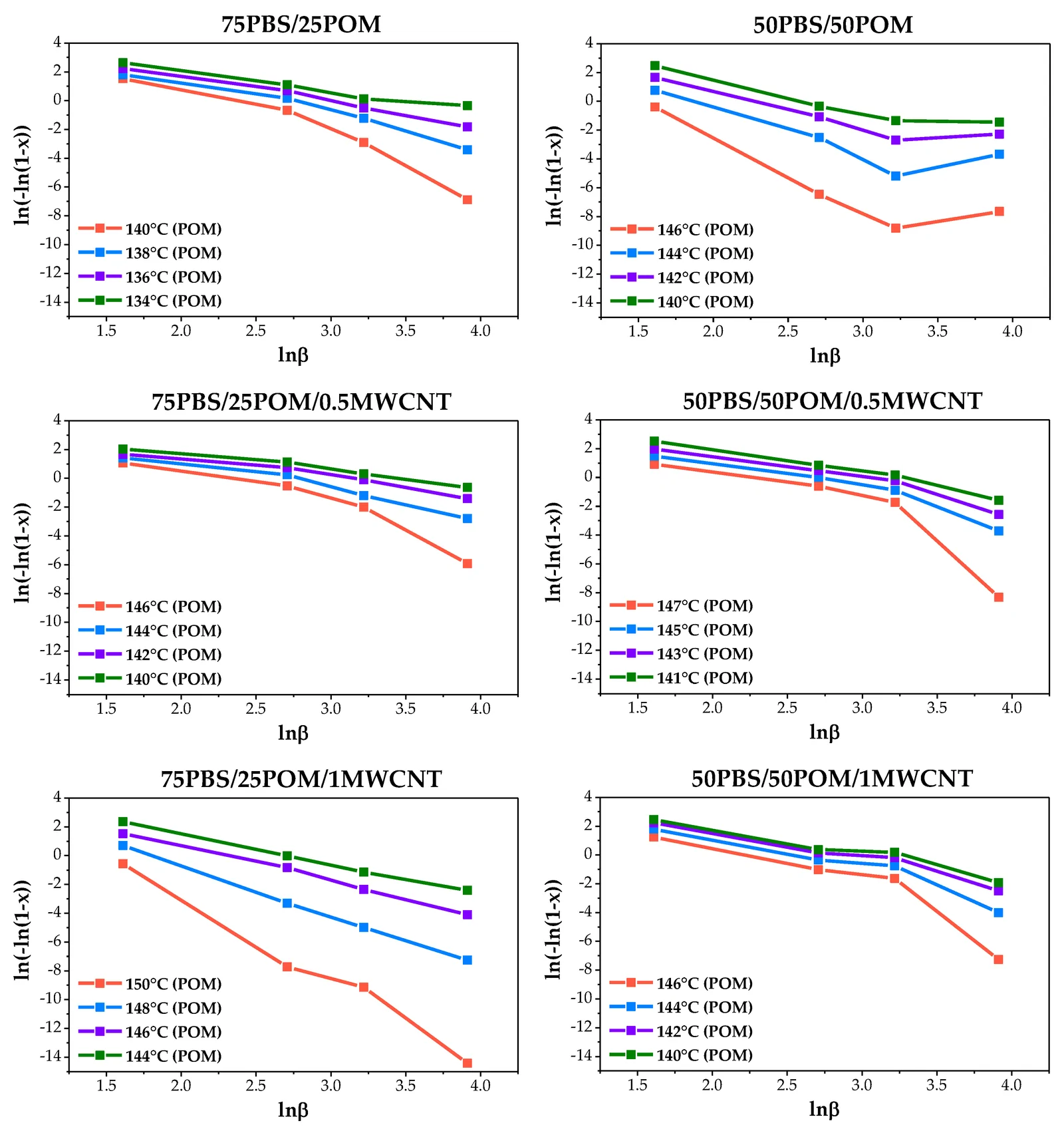
Ozawa curves of the POM phase in PBS/POM blends and PBS/POM/MWCNT nanocomposites.

**Figure 20:**
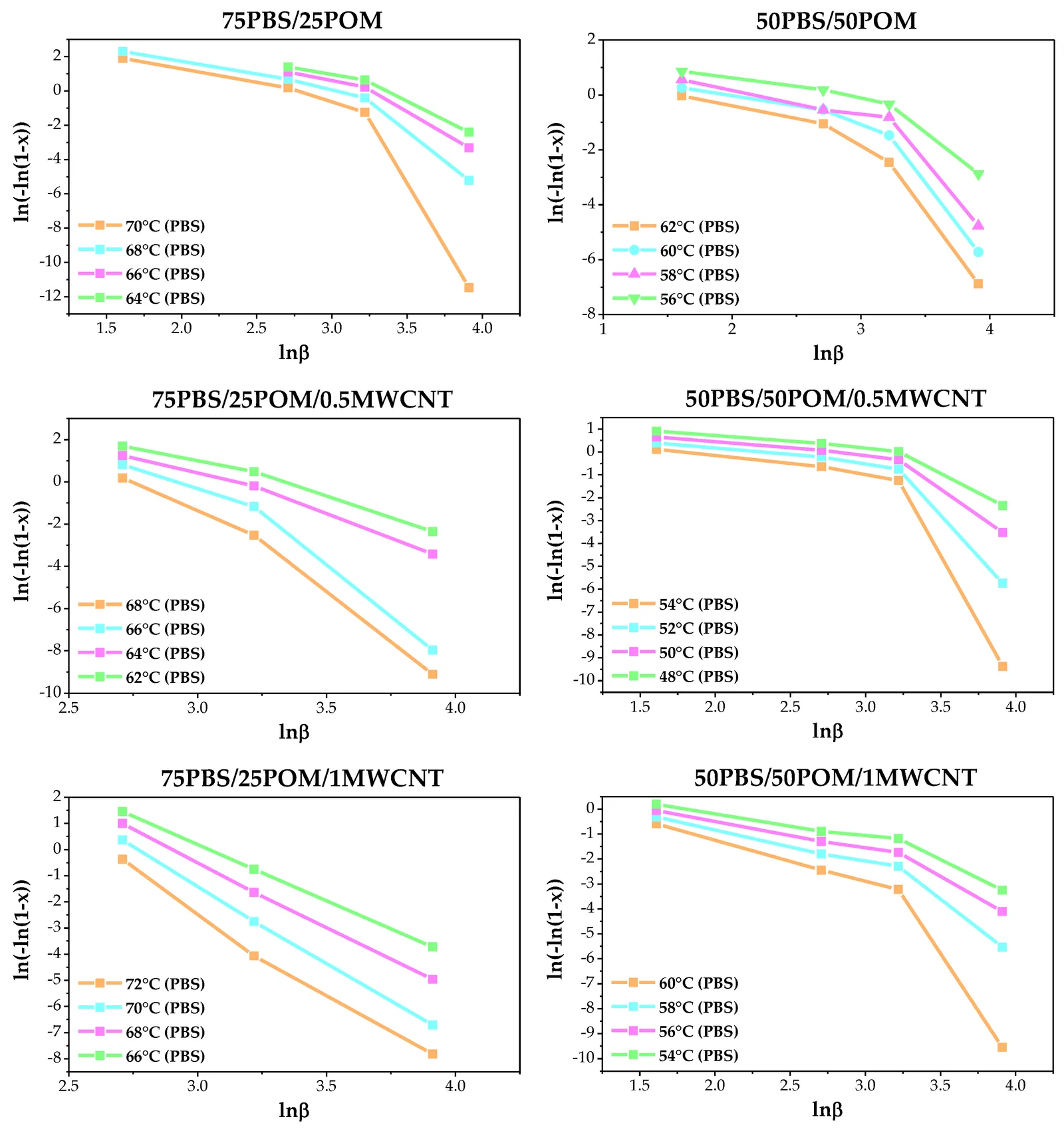
Ozawa curves of the PBS phase in PBS/POM blends and PBS/POM/MWCNT nanocomposites.

Figure 18 illustrates the Ozawa plots of neat PBS, neat POM, and their nanocomposites containing 0.5 and 1 wt.% MWCNT, whereas Figure 19 and Figure 20 presents the corresponding Ozawa plots for PBS/POM blend nanocomposites with different MWCNT loadings. It is evident from Figure 18, Figure 19 and Figure 20 that the Ozawa plots of neat polymers and all nanocomposite systems exhibit noticeable deviations from linearity. Since the Ozawa model assumes a linear relationship between ln[−ln(1 − *X(t)*)] and ln*β* at a given degree of crystallinity, the observed nonlinear behavior indicates that this model cannot adequately describe the non-isothermal crystallization kinetics of the investigated samples.

The deviation from linearity may be attributed to the complex nature of the crystallization process occurring during continuous cooling. Under different cooling conditions, nucleation and crystal growth proceed over varying temperature ranges, resulting in changes in the overall crystallization mechanism throughout the process. Consequently, the basic assumptions of the Ozawa approach are not fully satisfied for the PBS, POM, and PBS/POM-based nanocomposites examined in this study. Therefore, the Ozawa model was considered unsuitable for providing a quantitative description of their non-isothermal crystallization behavior [59,60].

To more accurately describe the non-isothermal crystallization process, Liu and Mo developed a combined Avrami–Ozawa model by incorporating both the cooling rate and crystallization time into the crystallization kinetics [61,62], as expressed by Equations (11) and (12).

(11) ln Z+n·ln t=ln K(T)−m·ln β

(12) ln β=ln F(T)−α·ln t

In Equation (12), *F(T)* represents the cooling rate required to attain a specific degree of crystallinity within a given unit of time. The parameter *α* is defined as the ratio of the Avrami exponent (*n*) to the Ozawa exponent (*m*), i.e., *α = n/m*. The value of *F(T)* is determined from the intercept of the linear relationship obtained by plotting *ln β* against *ln t*, while *α* is obtained from the slope of the corresponding linear equation. Furthermore, correlation coefficients approaching unity indicate a strong linear relationship between the parameters [59].

**Figure 21:**
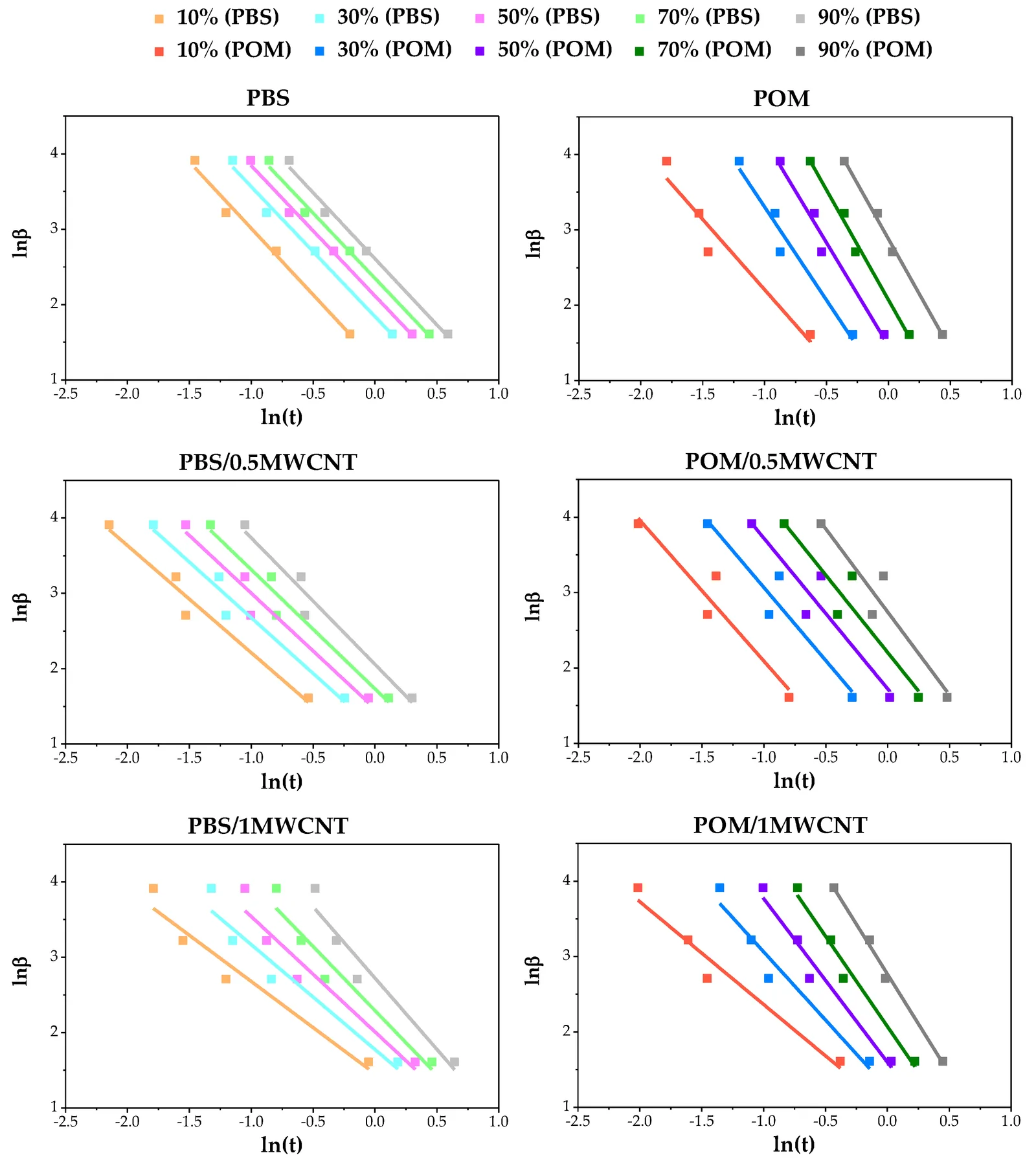
Combined Avrami–Ozawa curves of the PBS, POM, PBS/MWCNT, and POM/MWCNT samples.

**Figure 22:**
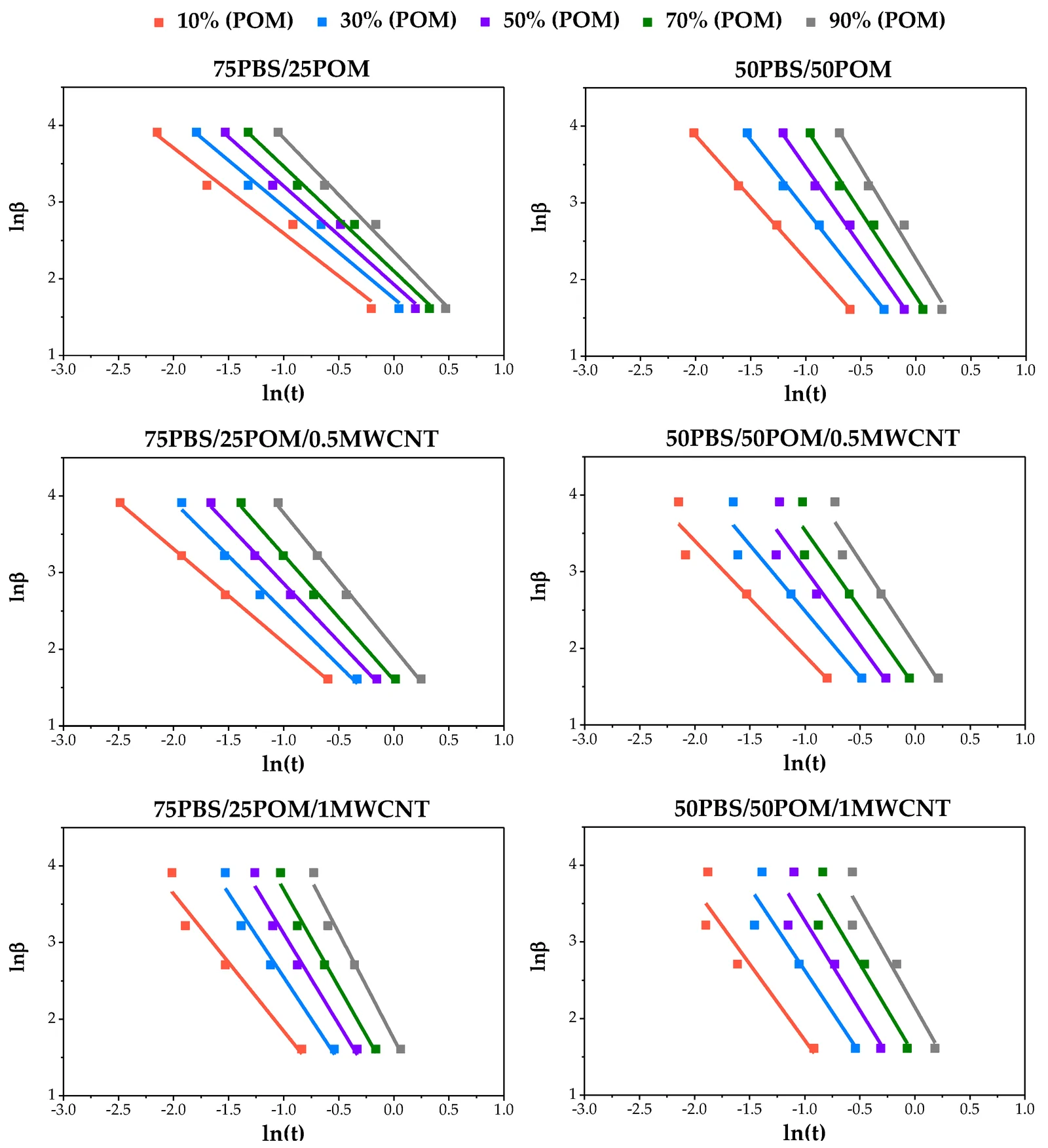
Combined Avrami–Ozawa curves of the POM phase in PBS/POM blends and PBS/POM/MWCNT nanocomposites.

**Figure 23:**
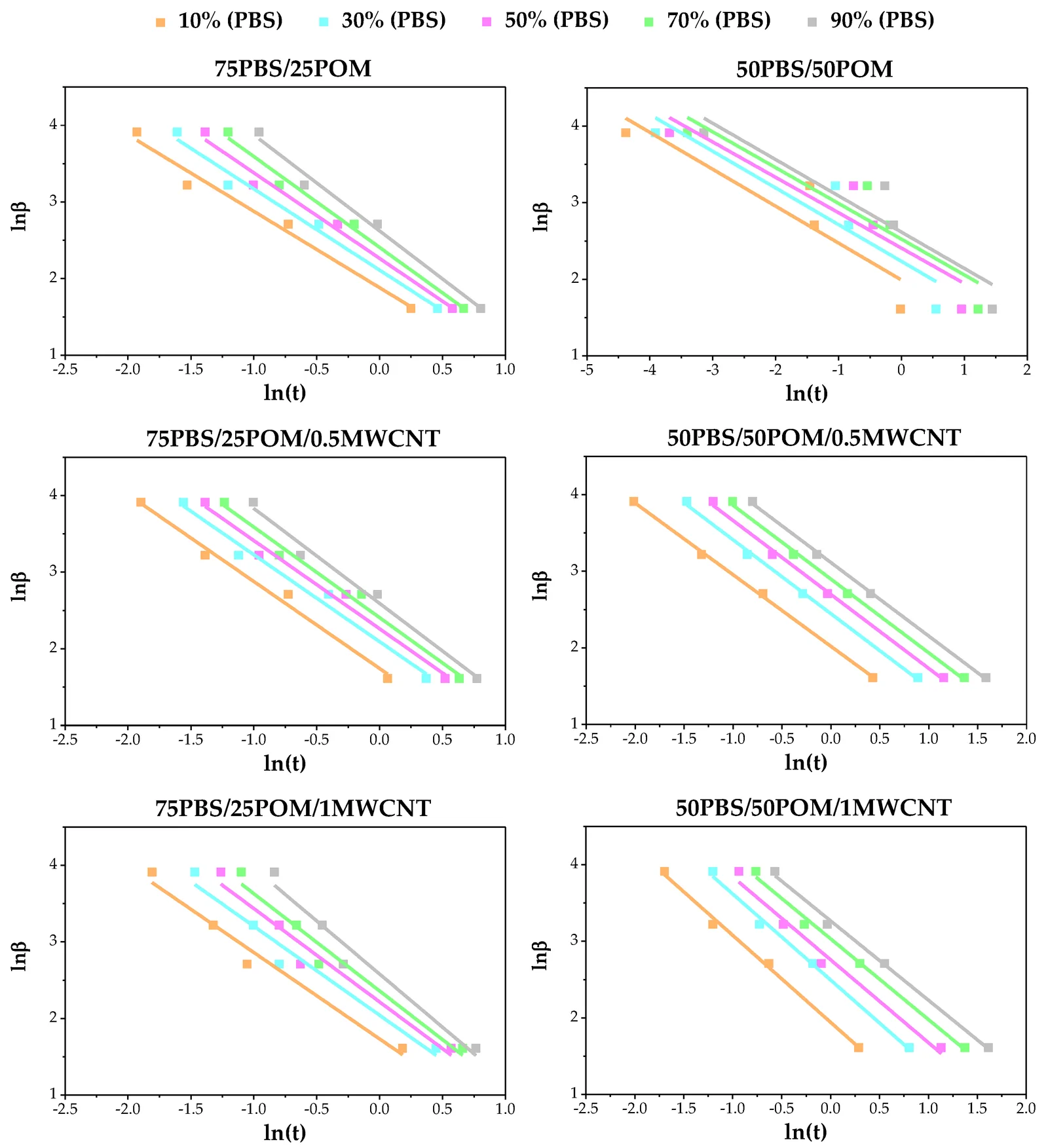
Combined Avrami–Ozawa curves of the PBS phase in PBS/POM blends and PBS/POM/MWCNT nanocomposites.

Figure 21 presents the combined Avrami–Ozawa plots for neat PBS, neat POM, and their nanocomposites containing different MWCNT loadings, while Figure 22 and Figure 23 show the corresponding plots for the PBS/POM/MWCNT blends. Table 10 summarizes the kinetic parameters obtained from the combined Avrami–Ozawa plots for neat POM, neat PBS, and the POM and PBS nanocomposites containing 0.5 and 1 wt.% MWCNT. The kinetic parameters derived from the combined Avrami–Ozawa curves for the PBS/POM/0.5MWCNT and PBS/POM/1MWCNT blends are presented in Table 11.

As shown in Figure 21, Figure 22 and Figure 23, plotting *ln β* against *ln t* at a given degree of crystallinity yielded a linear relationship, with correlation coefficients of *R*^2^ > 0.90. The 95% confidence intervals of the slopes ranged from ±0.01 to ±0.06, indicating a satisfactory linear fit of the combined Avrami–Ozawa model to the experimental data.

As seen in Table 10 and Table 11, *F(T)* values increased progressively with increasing relative crystallinity for neat PBS, neat POM, and PBS/POM blends of different compositions. Since a lower *F(T)* value corresponds to a higher crystallization rate, these results provide a quantitative indication of the crystallization kinetics of the investigated systems. For the PBS/MWCNT nanocomposites, the *F(T)* values obtained over the 10–90% relative crystallinity range were dependent on the MWCNT loading. The nanocomposites containing 0.5 wt.% MWCNT exhibited lower *F(T)* values than neat PBS, indicating an enhanced crystallization rate. Increasing the MWCNT content to 1 wt.% led to an increase in *F(T)*; nevertheless, the values remained below those of neat PBS, suggesting that the crystallization-promoting effect of MWCNT was retained, albeit to a lesser extent. In contrast, the POM/MWCNT nanocomposites generally exhibited higher *F(T)* values than neat POM, indicating that MWCNT incorporation hindered the overall crystallization kinetics of POM.

For the PBS/POM blends, incorporation of 25 wt.% POM resulted in higher *F(T)* values for both the PBS and POM phases, indicating a reduction in their respective crystallization rates. When the POM content was increased to 50 wt.%, the *F(T)* values of the PBS phase increased, whereas those of the POM phase decreased. This behavior suggests that, at the 50/50 composition, the crystallization of the POM phase was comparatively promoted, while that of the PBS phase was further retarded. For the 75PBS/25POM/MWCNT nanocomposites, the *F(T)* values of both the PBS and POM phases decreased relative to those of the corresponding 75PBS/25POM blend, indicating that MWCNT incorporation enhanced the crystallization kinetics of both phases. In the 50PBS/50POM/MWCNT nanocomposites, however, the *F(T)* values increased for the PBS phase but decreased for the POM phase compared with the corresponding 50PBS/50POM blend. Thus, MWCNT exerted a phase-dependent influence on the crystallization behavior of the blends. Overall, the results obtained from the combined Avrami–Ozawa analysis were generally consistent with the relative crystallinity–time data, particularly for neat PBS, neat POM, PBS/MWCNT and POM/MWCNT nanocomposites, as well as for the POM phase of the PBS/POM/MWCNT systems.

The *α* values obtained for PBS ranged from 1.73 to 1.76, whereas considerably higher values, ranging from 1.87 to 2.93, were determined for POM. The incorporation of MWCNT did not produce a pronounced variation in the *α* values of either PBS or POM, suggesting that MWCNT loading had a limited effect on the overall balance between the Avrami and Ozawa contributions represented by this parameter. In both PBS/POM samples, with and without MWCNT, generally lower *α* values were obtained than those determined for the corresponding neat polymers. Furthermore, the *α* values of the PBS phase decreased to below unity depending on the POM and MWCNT contents. This behavior indicates that MWCNT incorporation does not affect the Avrami and Ozawa exponents identically; rather, the *n* and *m* parameters are influenced to different extents, reflecting their distinct sensitivities to the nucleation and crystal growth processes.

In general, the non-isothermal crystallization behavior of polymeric systems is difficult to describe adequately using a single kinetic equation because multiple variables, including temperature, cooling rate, crystallization time, nucleation, and crystal growth mechanisms, are involved simultaneously. The principal advantage of the combined Avrami–Ozawa approach is therefore its ability to establish a relationship among cooling rate, temperature, crystallization time, and crystallization morphology, thereby providing a more comprehensive assessment of the non-isothermal crystallization kinetics [60]. This approach consequently offers valuable insight into the effects of polymer composition and MWCNT incorporation on the crystallization mechanisms of the investigated PBS/POM systems.

In this study, activation energies of samples were calculated by using Friedman method for each value of degree of crystallinity, ranging from 0.1 to 1. The Friedman equation can be expressed as follows:

(13) $$\mathrm{In}{\left(\frac{dX}{dt}\right)}_{X,t}\;=\;C\;-\;\frac{∆E_{X,t}}{{RT}_{X,t}}$$

where *dXt/dt* is the instantaneous crystallization rate as a function of time for a given value of the relative crystallinity (*X_t_*), R is the universal gas constant, and Δ*E_X,t_* is the effective energy barrier of the process for a given value of *X_t_*, and C is a constant. By plotting the left hand side of Equation 13 with respect to *1/T_X,t_,* a straight line must be obtained with a slope of *−*Δ*E_X_/R*. For this purpose, *dX_t_/dt* versus *1/T_X,t_* were plotted for each cooling rate.

**Figure 24:**
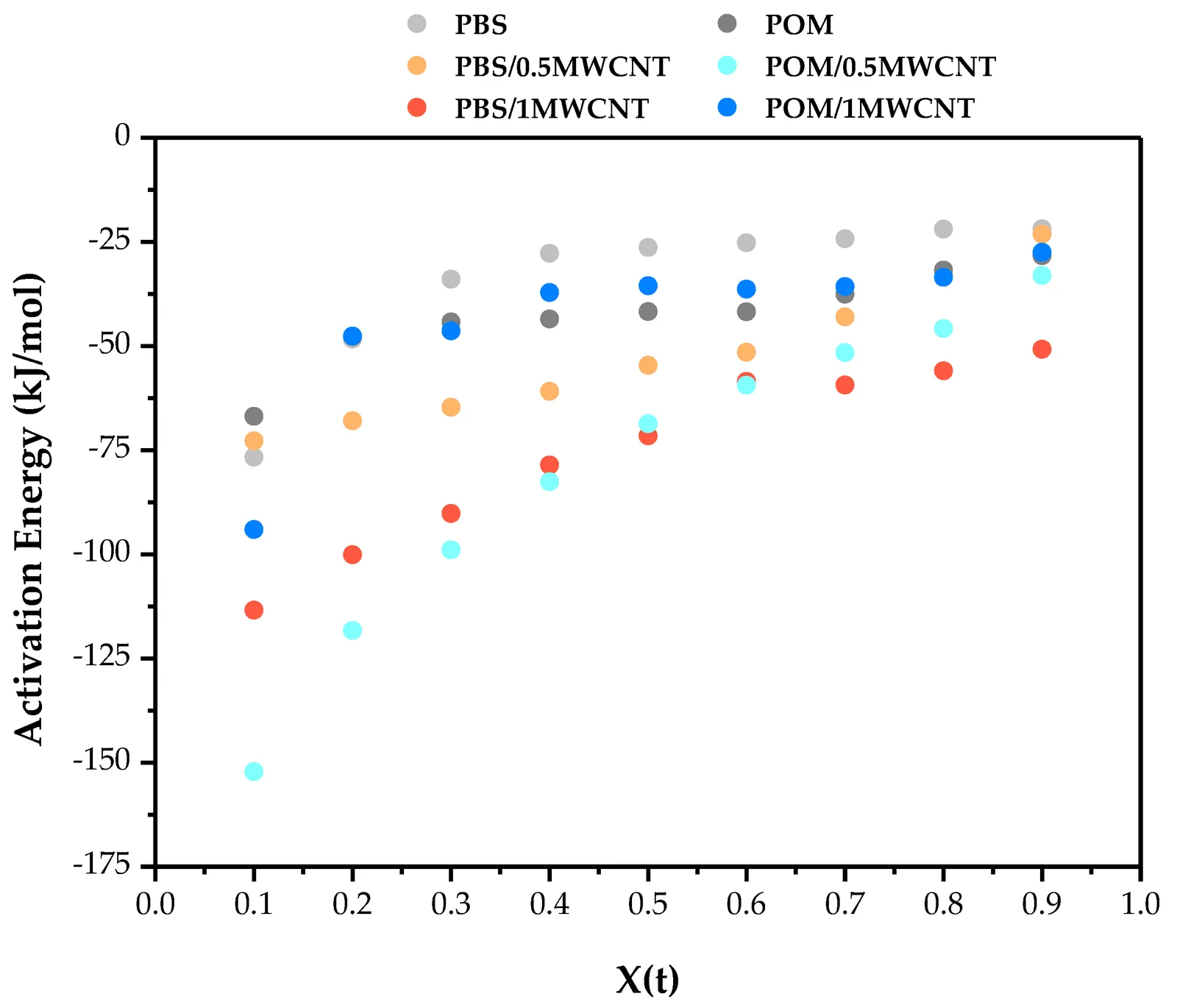
Activation energy as a function of relative crystallinity for PBS, POM, PBS/MWCNT, and POM/MWCNT samples.

**Figure 25:**
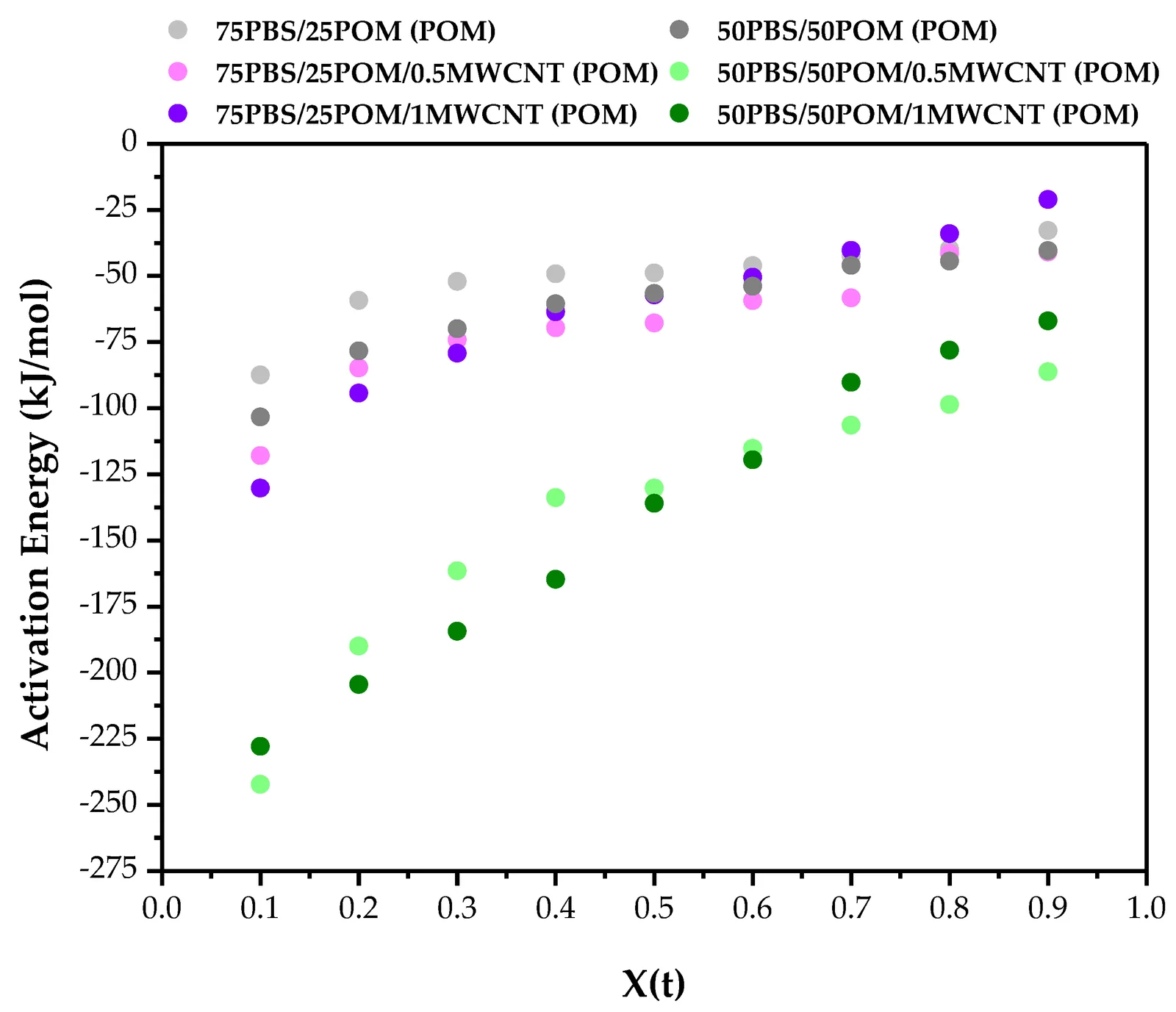
Activation energy as a function of relative crystallinity for the POM phase in PBS/POM blends and PBS/POM/MWCNT nanocomposites.

**Figure 26:**
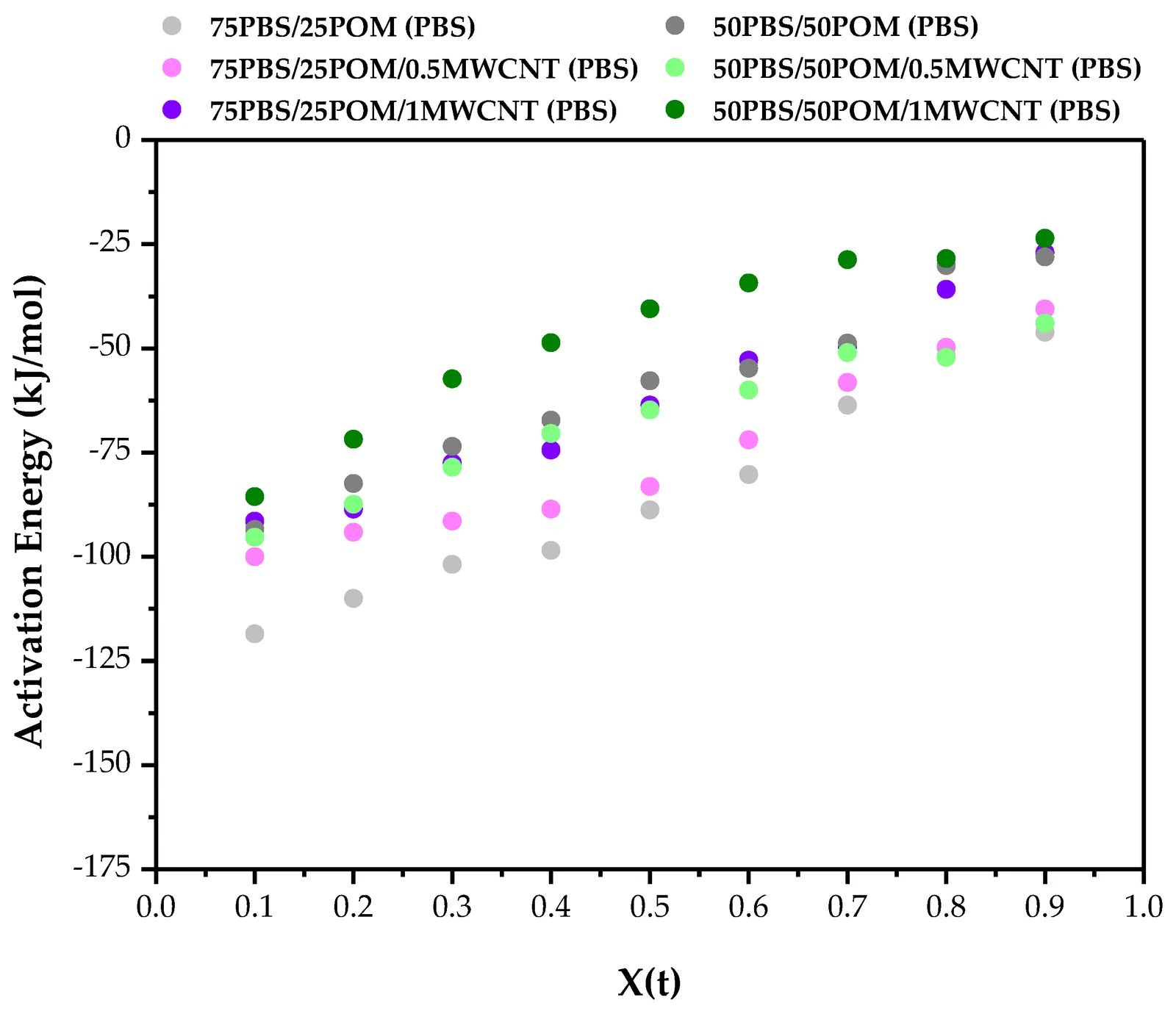
Activation energy as a function of relative crystallinity for the PBS phase in PBS/POM blends and PBS/POM/MWCNT nanocomposites.

Figure 24 presents the changes in activation energy as a function of relative crystallinity for neat PBS, neat POM, POM/MWCNT, and PBS/MWCNT nanocomposites, while Figure 25 and Figure 26 show the corresponding results for PBS/POM blends and PBS/POM/MWCNT nanocomposites, respectively. For neat PBS, the addition of MWCNT was found to decrease the activation energy at any given relative crystallinity. Furthermore, the decrease in activation energy continued with increasing MWCNT content. In the case of POM/MWCNT nanocomposites, the activation energy decreased at low MWCNT contents, whereas it increased at higher MWCNT loadings. In addition, as expected for all samples, the activation energy was relatively low at low relative crystallinity values, corresponding to the formation of embryonic crystals, and shifted toward higher values with increasing relative crystallinity as the embryonic crystals progressively developed and matured.

The Friedman curves obtained for the POM phase of the PBS/POM blends and PBS/POM/MWCNT nanocomposites are presented in Figure 25. As can be seen from Figure 25, the energy barrier required for crystallization of the POM phase decreased with increasing POM content in the PBS/POM blends. This behavior can be attributed to the nucleating effect of PBS on POM crystallization. In the presence of MWCNT, the activation energy values of the POM phase in the 75PBS/25POM blend were generally lower than those of the corresponding blend without MWCNT at a given relative crystallinity. A similar trend was also observed for the 50PBS/50POM blend.

Figure 26 presents the activation energy as a function of relative crystallinity for the PBS phase of the PBS/POM/MWCNT nanocomposites. As shown in Figure 26, the incorporation of 0.5 and 1 wt.% MWCNT into the 75PBS/25POM blend resulted in higher activation energy values than those of the 75PBS/25POM blend without MWCNT. This behavior can be attributed to the preferential nucleation of POM by MWCNT, which suppresses the crystallization of the PBS phase. For the 50PBS/50POM blends, the addition of MWCNT resulted in a further increase in the activation energy. This finding indicates that when the POM content reaches 50 wt.%, the crystallization of the PBS phase is suppressed to a greater extent.

### 3.6. Polarized Optical Microscopy

Polarized optical microscopy analyses were performed to investigate the crystal morphologies of the PBS, POM, PBS/POM blends, POM/MWCNT, PBS/MWCNT, and PBS/POM/MWCNT nanocomposites. For polarized optical microscopy analyses, all samples were heated from 25 to 200 °C at a rate of 30 °C/min, held at 200 °C for 5 min, and then cooled to 30 °C at a rate of 1 °C/min. POM images were obtained from the recorded videos in the temperature range of 150–80 °C to examine the formation and growth behavior of embryonic crystals.

**Figure 27:**
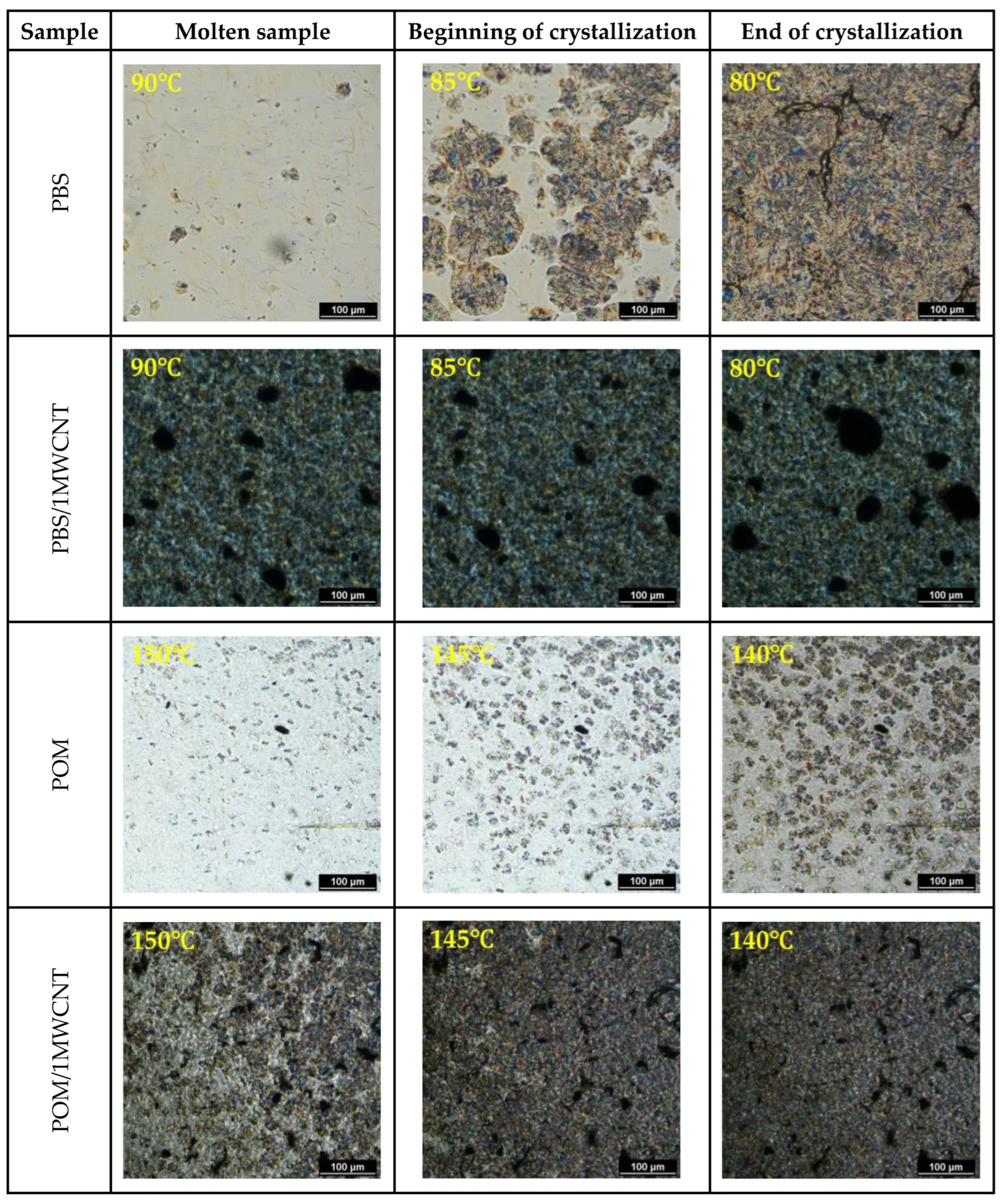
POM images of the PBS, POM, PBS/MWCNT, and POM/MWCNT nanocomposites.

Figure 27 presents the polarized optical microscopy images of neat PBS, neat POM, and PBS/MWCNT and POM/MWCNT nanocomposites containing 1 wt.% MWCNT. The crystallization of PBS started at approximately 95 °C and was completed at around 80 °C. With the incorporation of 1 wt.% MWCNTs, the crystallite size of PBS decreased, while its crystalline density increased. This behavior suggests that, in the presence of MWCNTs, PBS crystallization proceeds via a higher density of nucleation sites, indicating that MWCNTs function as effective nucleating agents for PBS. In contrast, neat POM initiated melt crystallization at approximately 150 °C and demonstrated a higher crystalline density than PBS, consistent with its intrinsically high degree of crystallinity. Furthermore, the addition of MWCNTs was found to further enhance the nucleation density of POM. This showed that MWCNT acted as a nucleating agent for POM.

**Figure 28:**
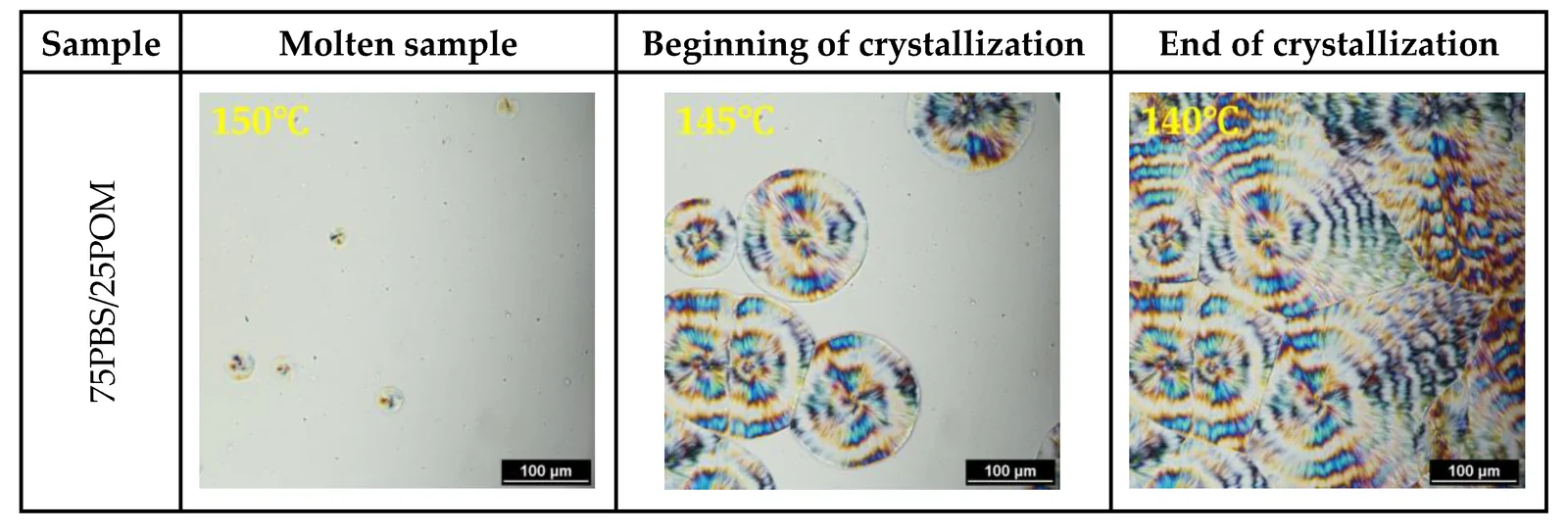

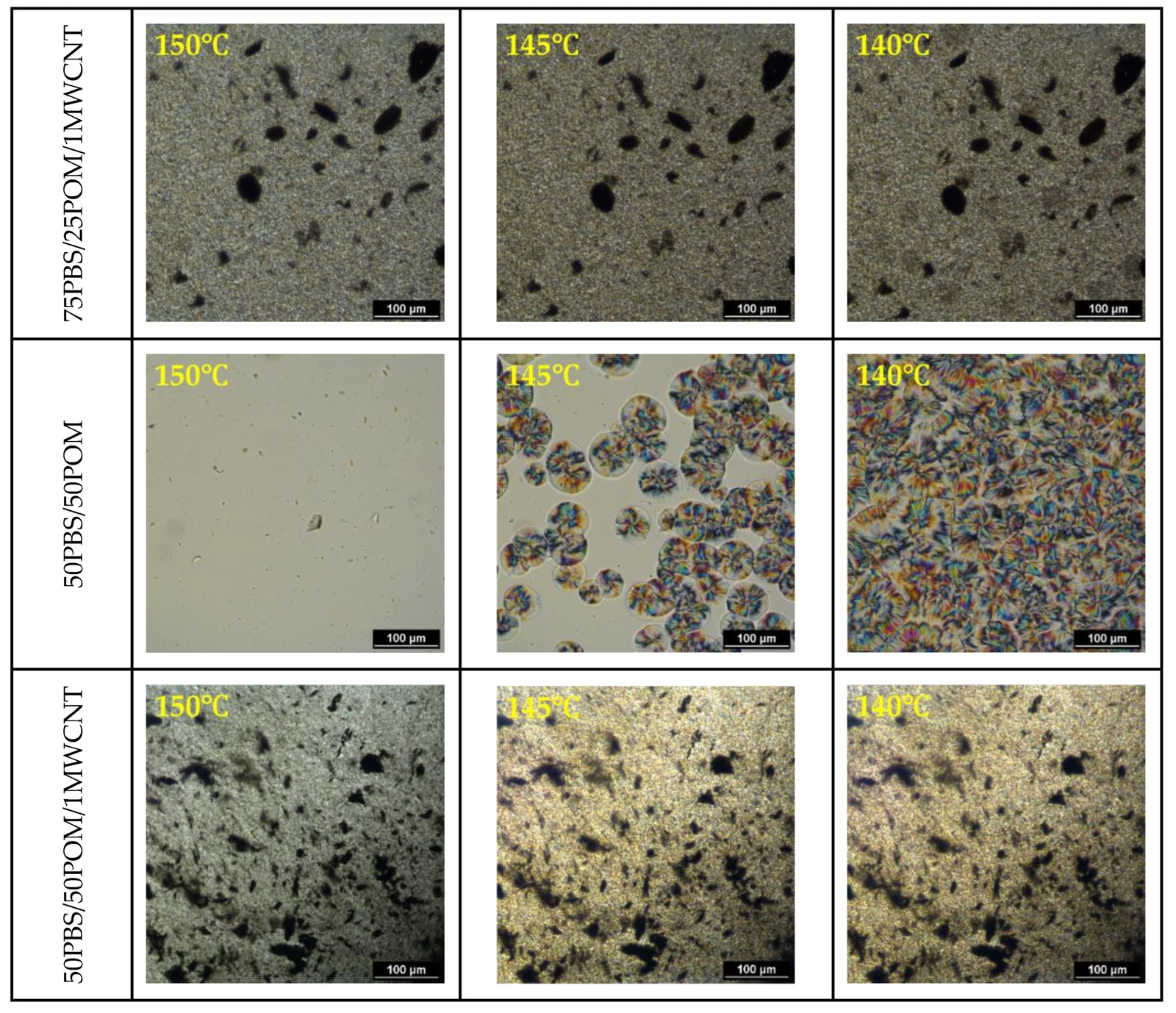
POM images of the PBS/POM blends and PBS/POM/MWCNT nanocomposites.

In PBS/POM blends with different compositions, owing to the large difference in melting temperatures between the two polymers, PBS remained in the molten state, whereas POM began to crystallize. As seen from Figure 28, POM crystallized in the form of spherical (3D) spherulites. In the 75PBS/25POM blend, POM spherulites formed at 150 °C, and crystallization was completed at 140 °C. In the 50PBS/50POM blend, a higher nucleation density was observed, and crystallization occurred at comparatively lower temperatures. It was also found that the spherulite size of POM decreased with increasing PBS content, whereas the spherulite density increased.

The incorporation of MWCNTs markedly influenced the crystal morphology of all blends. At 1 wt.% MWCNT, crystallization occurred at higher temperatures, accompanied by an increase in spherulite density and a reduction in spherulite size, indicating that MWCNTs serve as effective nucleating agents within the system. Moreover, as the temperature approached the crystallization range of PBS, POM crystals dominated the observed field, hindering clear identification of the PBS phase in the images; consequently, images acquired at lower temperatures were excluded for the PBS/POM blends. These observations are in good agreement with the non-isothermal crystallization results and further confirm the nucleating and crystallization-accelerating effects of MWCNTs.

### 3.7. X-Ray Diffaction

**Figure 29:**
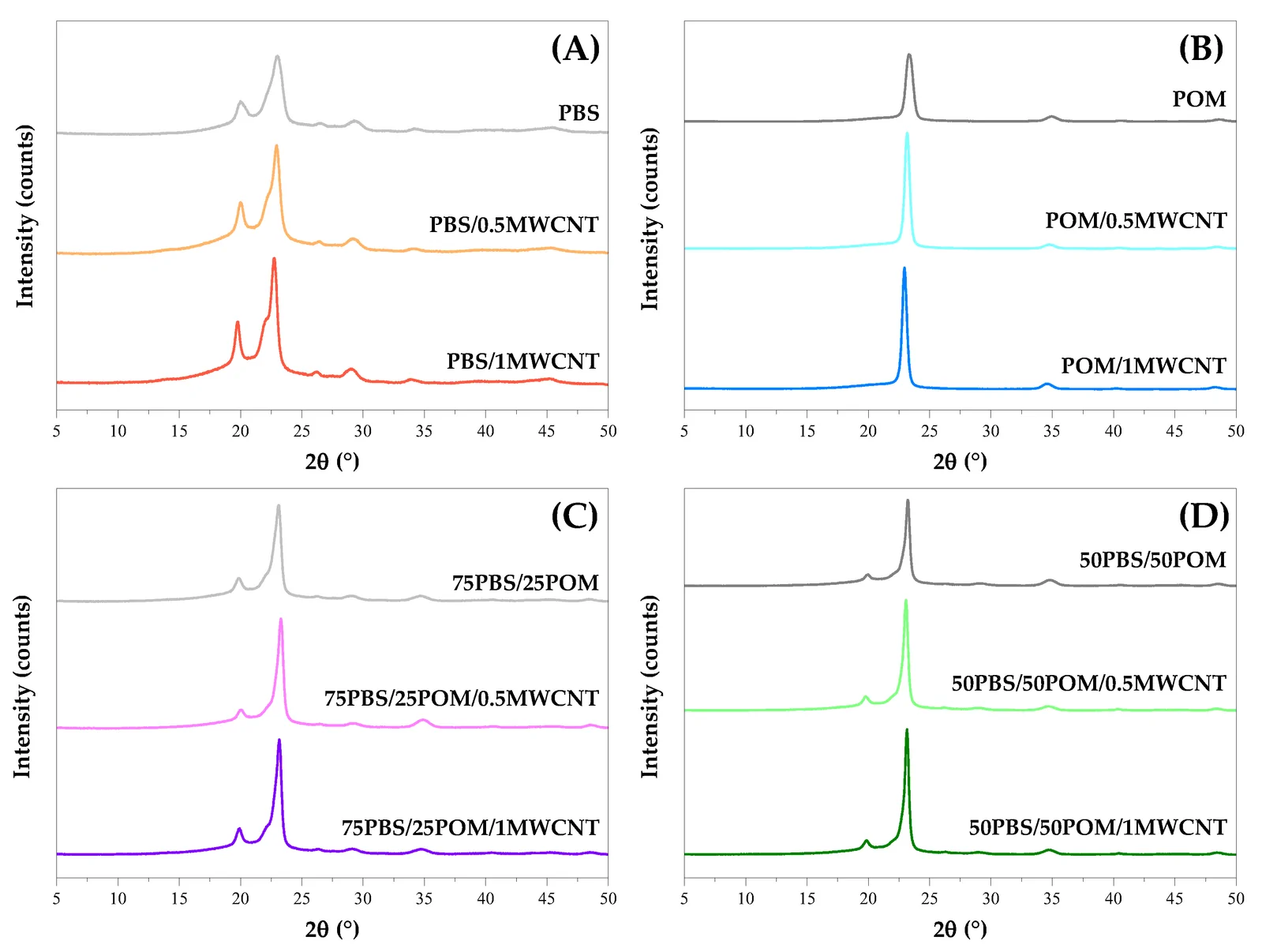
The XRD patterns of; (**A**) PBS and PBS/MWCNT, (**B**) POM and POM/MWCNT, (**C**) 75PBS/25POM and 75PBS/25POM/MWCNT, (**D**) 50PBS/50POM and 50PBS/50POM/MWCNT samples.

The crystallographic characteristics of the samples were investigated by X-ray diffraction (XRD). As shown in Figure 29, neat PBS exhibits diffraction peaks at 2θ = 20.03°, 21.08°, 22.95°, 25.36°, and 29.09°, which can be assigned to the (111)/(002), (012), (110), (121), and (111) planes of the α-crystalline form, respectively [63]. In the case of neat POM, the diffraction peaks observed at 2θ = 23.16°, 34.74°, 40.44°, and 48.40° are attributed to the (100), (105), (110), and (115) crystallographic planes, respectively [64,65].

The PBS/MWCNT and POM/MWCNT nanocomposites exhibit XRD profiles generally consistent with those of their corresponding neat polymers, indicating that the incorporation of MWCNT does not alter the fundamental crystal structures of PBS or POM. Nevertheless, variations in the intensities of specific diffraction peaks are observed upon MWCNT addition. In particular, the intensity of the (100) reflection of POM and those of the (111)/(002) and (012) reflections of PBS increase in the presence of MWCNT. These changes suggest that MWCNT promotes the crystallization of both POM and PBS, resulting in an increase in their degree of crystallinity within the nanocomposites.

**Figure 30:**
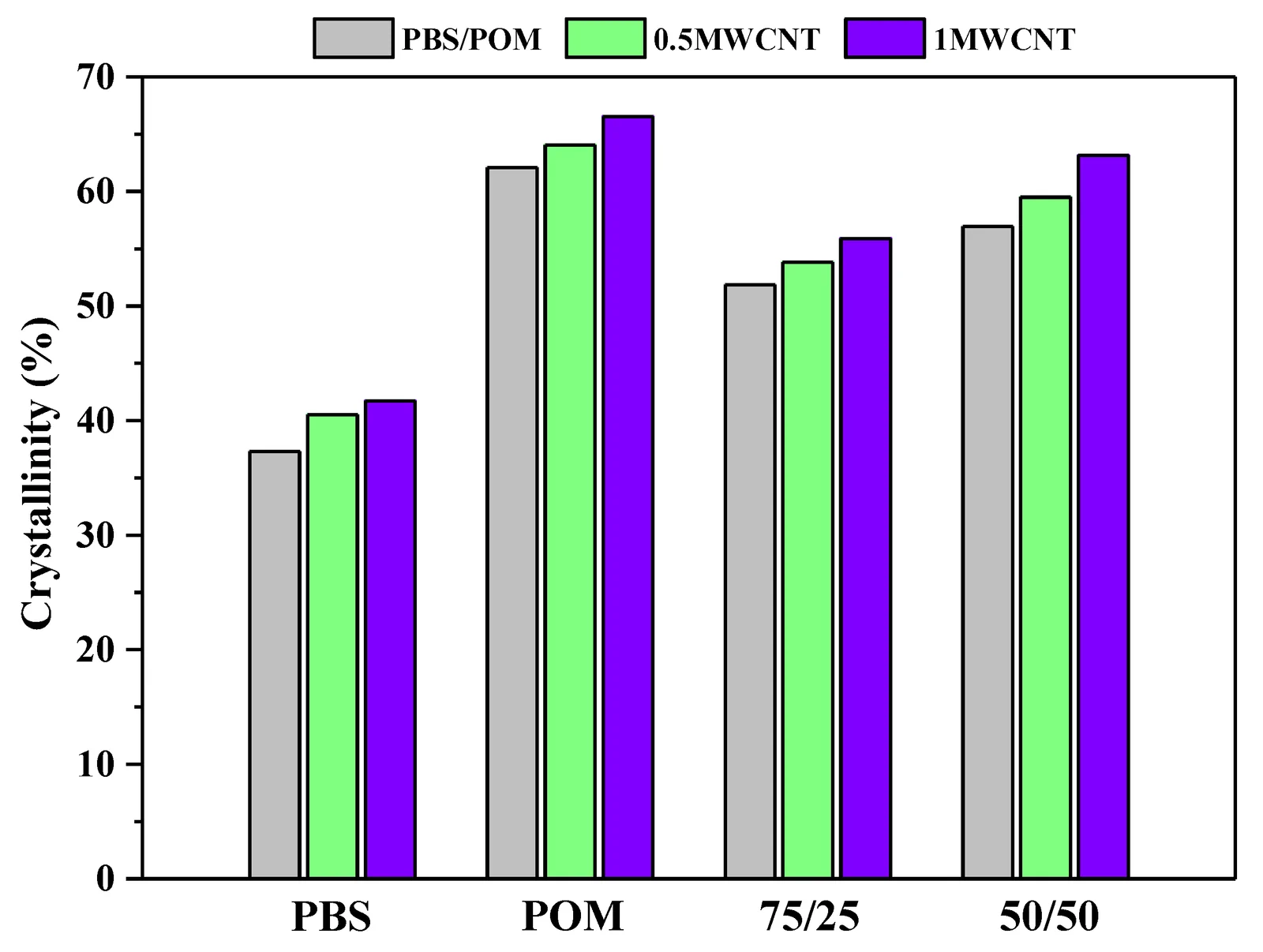
The percent crystallinity of samples.

When the XRD patterns of the PBS/POM blends were examined, the intensities of the (111)/(002) and (111) planes of the α-crystalline form were found to decrease in the presence of 25 wt.% POM. This decrease became more pronounced as the POM content was increased to 50 wt.%, with the (111) reflection of the α-crystalline form being almost completely suppressed. These results indicate that the incorporation of POM significantly alters the crystalline ordering of PBS. The XRD results further demonstrate that the addition of MWCNT increases the overall crystallinity of the PBS/POM blends, and this increase is mainly attributed to the enhanced crystallinity of the POM phase (Figure 30).

## 4. Conclusions

This research focused on analyzing the mechanical, thermal, structural, morphological, and crystallization attributes of PBS/POM blends and their nanocomposites reinforced with MWCNT. The FTIR analysis revealed interactions between PBS and POM in PBS/POM blends. Moreover, it was found that physical interactions between MWCNT and PBS or POM can significantly enhance interfacial wetting and mechanical interlocking without altering the chemical integrity of the matrices. The SEM images showed a relatively continuous morphology of the PBS/POM blends, with no pronounced micron-scale MWCNT agglomerates readily discernible within the examined regions. These observations suggest a relatively favorable distribution of MWCNTs at the spatial scale accessible by SEM, while the underlying PBS/POM blends exhibited a partially miscible morphology without obvious micron-scale phase separation. The observed MWCNT distribution, together with the interfacial characteristics of the blend, may contribute to the overall mechanical and thermomechanical performance of the PBS/POM/MWCNT nanocomposites. From a mechanical standpoint, incremental additions of both the POM content and the MWCNT loading yielded a concomitant increase in tensile strength and elastic modulus. The tensile strength of neat PBS increased from 21.7 ± 0.4 MPa to 30.4 ± 0.1 MPa with the addition of 1 wt.% MWCNT, corresponding to an approximately 40% improvement. Conversely, a typical trade-off was observed as the elongation at break and impact strength progressively decreased, reflecting a transition toward a more brittle material behavior. Furthermore, DMA indicated that MWCNT addition improved the storage modulus and induced a slight upward shift in the glass transition temperature. Concurrently, the reduction in tan δ peak intensity suggests a decrease in the damping response, which may be associated with restricted segmental mobility and possible changes in the interfacial interactions between the constituent phases. Non-isothermal crystallization and polarized optical microscopy analyses collectively revealed that MWCNTs functioned as highly effective heterogeneous nucleating agents, elevating the crystallization temperature and accelerating crystallization kinetics while inducing the formation of smaller, more densely packed spherulitic structures. For instance, the crystallization onset temperature increased from 88.20 °C to 98.11 °C at a cooling rate of 5 °C/min upon addition of 1 wt.% MWCNT, corresponding to a 10 °C increase for neat PBS. Ultimately, these comprehensive findings demonstrated that the enhanced mechanical and thermal profiles of the PBS/POM/MWCNT ternary systems stem from a synergistic combination of structural compatibility, robust interfacial interactions, and altered crystallization behavior.

## Figures and Tables

**Table 1:** Surface energy components (mJ/m^2^) of the probe liquids [35].

Probe Liquids	$\gamma_{i}^{TOT}$	$\gamma_{i}^{LW}$	$\gamma_{i}^{AB}$	$\gamma_{i}^{-}$	$\gamma_{i}^{+}$
Diiodomethane	50.80	50.80	-	-	-
Ethylene Glycol	48.00	29.00	19.00	30.10	3.00
Formamide	58.00	39.00	19.00	39.60	2.30

**Table 2:** Average contact angles of probe liquids on the surface of PBS and POM.

Polymer	Contact Angle (θ)		
	Diiodomethane	Ethylene Glycol	Formamide
PBS	50.80	50.80	-
POM	48.00	29.00	19.00

**Table 3:** The surface energies (mJ/m^2^) of neat polymers and MWCNT.

Sample	$\gamma_{S}^{-}$	$\gamma_{S}^{+}$	$\gamma_{S}^{AB}$	$\gamma_{S}^{LW}$	$\gamma_{S}^{TOT}$
PBS	84.64	12.33	64.60	41.71	106.31
POM	1.65	0.21	1.18	38.05	39.23
MWCNT	12.62	2.17	10.47	35.67	46.14

**Table 4:** Interfacial energies between polymer–MWCNT, PBS-POM and the wetting parameter.

$\gamma_{\mathrm{PBS}\text{-}\mathrm{MWCNT}}$	23.29 mJ/m^2^
$\gamma_{\mathrm{POM}\text{-}\mathrm{MWCNT}}$	4.66 mJ/m^2^
$\gamma_{\mathrm{PBS}\text{-}\mathrm{POM}}$	48.40 mJ/m^2^
$\omega_{a}$	0.38

**Table 5:** Mechanical properties of samples.

Sample	Tensile Strength (MPa)	Elongation at Break (%)	Young’s Modulus (MPa)	Impact Strength (kJ/m^2^)
PBS	21.7 ± 0.4	21.8 ± 1.1	766.1 ± 43.6	5.6 ± 0.1
PBS/0.5MWCNT	23.8 ± 0.9	13.5 ± 1.3	814.0 ± 35.2	5.0 ± 0.5
PBS/1MWCNT	30.4 ± 0.1	10.3 ± 2.2	893.6 ± 45.9	4.3 ± 0.4
POM	48.7 ± 1.3	50.0 ± 1.9	2827.5 ± 198.6	6.6 ± 0.3
POM/0.5MWCNT	60.6 ± 1.4	26.6 ± 2.7	3179.8 ± 143.7	5.5 ± 0.7
POM/1MWCNT	62.8 ± 0.9	16.5 ± 1.3	3532.3 ± 56.4	5.0 ± 0.1
75PBS/25POM	26.5 ± 1.2	35.3 ± 0.4	1322.7 ± 134.6	4.9 ± 0.3
75PBS/25POM/0.5MWCNT	31.6 ± 2.0	30.6 ± 0.2	1407.9 ± 122.1	4.6 ± 0.5
75PBS/25POM/1MWCNT	38.1 ± 0.8	17.7 ± 1.4	1501.6 ± 178.6	4.2 ± 0.1
50PBS/50POM	38.8 ± 0.2	42.6 ± 0.4	1514.6 ± 49.5	5.3 ± 0.2
50PBS/50POM/0.5MWCNT	45.3 ± 3.9	35.3 ± 0.3	1627.4 ± 204.4	5.0 ± 0.3
50PBS/50POM/1MWCNT	47.2 ± 2.7	29.0 ± 0.3	1798.7 ± 99.5	4.6 ± 0.3

**Table 6:** Melt crystallization onset, crystallization peak and crystallization endset temperatures and crystallization half-time values of PBS, POM, PBS/MWCNT and POM/MWCNT samples obtained from the cooling segment.

Sample	β (°C/min)	T_c,onset_ (°C)	T_c,peak_ (°C)	T_c,endset_ (°C)	t_1/2_ (min)
PBS	5	88.20	81.74	77.26	1.35
	15	83.14	73.75	66.33	0.71
	25	79.10	69.24	59.43	0.49
	50	73.30	59.75	45.70	0.37
PBS/0.5MWCNT	5	96.57	93.04	87.69	0.95
	15	93.41	89.07	81.30	0.36
	25	90.47	85.10	73.67	0.35
	50	85.34	78.66	62.29	0.21
PBS/1MWCNT	5	98.11	94.58	88.42	1.37
	15	93.70	89.52	69.80	0.53
	25	92.09	87.11	68.46	0.43
	50	86.07	75.06	51.57	0.35
POM	5	149.45	148.03	141.42	1.03
	15	147.66	144.25	131.07	0.53
	25	145.97	141.37	119.84	0.47
	50	144.15	130.80	103.32	0.33
POM/0.5MWCNT	5	151.55	147.07	140.32	1.01
	15	148.76	144.60	130.81	0.58
	25	147.59	135.67	117.93	0.51
	50	145.45	134.79	110.04	0.36
POM/1MWCNT	5	151.69	148.99	139.66	0.96
	15	149.27	143.06	128.43	0.59
	25	147.10	135.79	116.46	0.55
	50	145.72	126.89	101.27	0.41

**Table 7:** Melt crystallization onset, crystallization peak and crystallization endset temperatures and crystallization half-time values of PBS/POM and PBS/POM/MWCNT samples obtained from the cooling segment.

Sample	β (°C/min)	T_c,onset_ (°C)		T_c,peak_ (°C)		T_c,endset_ (°C)		t_1/2_ (min)	
		PBS	POM	PBS	POM	PBS	POM	PBS	POM
75PBS/25POM	5	84.46	148.02	79.10	143.94	74.26	139.51	1.78	1.21
	15	78.22	145.38	71.72	139.50	63.68	131.73	0.71	0.61
	25	76.16	143.11	68.15	136.51	57.96	123.95	0.37	0.33
	50	69.13	140.17	59.46	132.91	44.09	115.14	0.25	0.21
75PBS/25POM/ 0.5MWCNT	5	84.68	152.29	78.21	149.61	74.03	144.51	1.68	0.86
	15	79.90	150.23	69.23	146.76	62.14	137.75	0.76	0.41
	25	72.93	148.17	64.96	144.47	53.63	130.41	0.38	0.28
	50	62.65	146.51	55.18	140.14	43.35	123.51	0.25	0.19
75PBS/25POM/ 1MWCNT	5	86.29	152.65	79.95	150.12	75.80	145.38	1.75	0.71
	15	79.32	149.20	75.61	146.75	72.05	142.08	0.53	0.39
	25	75.50	148.18	65.42	142.63	52.38	130.56	0.45	0.28
	50	66.53	145.90	57.32	135.07	41.00	120.59	0.28	0.20
50PBS/50POM	5	75.79	149.42	61.70	146.12	47.97	141.50	2.60	0.90
	15	69.02	146.56	62.23	141.47	44.52	127.03	0.63	0.54
	25	65.27	144.27	58.05	137.70	40.49	119.84	0.46	0.39
	50	58.58	141.17	49.11	131.13	28.89	106.34	0.25	0.29
50PBS/50POM/ 0.5MWCNT	5	69.26	152.87	55.53	150.63	44.16	144.58	3.16	0.76
	15	65.74	150.89	53.24	148.11	39.75	134.08	0.96	0.42
	25	63.46	149.11	50.72	145.85	34.91	129.63	0.55	0.28
	50	52.62	146.63	39.62	137.61	25.69	113.67	0.30	0.27
50PBS/50POM/ 1MWCNT	5	73.66	153.09	56.56	150.52	43.35	144.72	3.08	0.75
	15	64.99	150.45	51.05	150.82	35.42	132.83	1.10	0.48
	25	62.99	149.04	50.61	144.70	33.15	123.36	0.61	0.31
	50	54.93	146.17	38.93	131.89	25.01	109.42	0.39	0.33

**Table 8:** Avrami kinetic parameters of PBS, POM, PBS/MWCNT and POM/MWCNT nanocomposites at different cooling rates.

Sample	β (°C/min)	*n*	Z (min^−n^)	R^2^	t_0.5_ (min)	1/t_0.5_ (min^−1^)
PBS	5	2.10	0.05	0.99	3.50	0.29
	15	2.09	0.17	1.00	1.96	0.51
	25	2.23	0.71	1.00	0.99	1.01
	50	2.43	1.99	1.00	0.65	1.54
PBS/0.5MWCNT	5	2.24	0.09	1.00	2.49	0.40
	15	2.00	0.77	0.99	0.95	1.05
	25	2.80	4.43	1.00	0.52	1.94
	50	2.35	7.73	0.98	0.36	2.79
PBS/1MWCNT	5	2.14	0.05	1.00	3.52	0.28
	15	2.34	0.72	1.00	0.98	1.02
	25	2.46	1.95	1.00	0.66	1.52
	50	2.80	9.81	1.00	0.39	2.57
POM	5	2.15	0.01	0.95	7.18	0.14
	15	3.05	2.96	1.00	0.62	1.61
	25	2.94	3.54	0.94	0.57	1.74
	50	2.45	11.89	0.98	0.31	3.19
POM/0.5MWCNT	5	1.86	0.06	1.00	3.73	0.27
	15	2.70	2.07	0.96	0.67	1.50
	25	2.69	2.30	0.97	0.64	1.56
	50	2.53	10.85	1.00	0.34	2.96
POM/1MWCNT	5	2.49	0.06	1.00	2.72	0.37
	15	2.38	1.32	1.00	0.76	1.31
	25	2.38	1.69	1.00	0.69	1.45
	50	2.52	8.76	1.00	0.37	2.74

**Table 9:** Avrami kinetic parameters of PBS/POM blends and PBS/POM/MWCNT nanocomposites at different cooling rates.

Sample	β (°C/min)	*n*		Z (min^−n^)		R^2^		t_0.5_ (min)		1/t_0.5_ (min^−1^)	
		PBS	POM	PBS	POM	PBS	POM	PBS	POM	PBS	POM
75PBS/25POM	5	1.98	1.96	0.01	0.01	1.00	0.99	8.51	11.20	0.12	0.09
	15	2.05	2.58	0.08	0.01	1.00	0.99	2.87	6.64	0.35	0.15
	25	2.20	2.78	1.82	8.47	1.00	1.00	0.64	0.41	1.55	2.46
	50	2.52	2.99	10.54	55.01	1.00	1.00	0.34	0.23	2.94	4.32
75PBS/25POM/ 0.5MWCNT	5	2.03	2.18	0.02	0.01	1.00	1.00	5.73	6.99	0.17	0.14
	15	2.13	2.31	0.26	0.02	1.00	1.00	1.58	4.64	0.63	0.22
	25	2.67	2.51	2.17	9.22	1.00	0.98	0.65	0.36	1.53	2.80
	50	3.48	2.26	46.18	25.64	1.00	1.00	0.30	0.20	3.34	4.94
75PBS/25POM/ 1MWCNT	5	2.10	1.97	0.03	0.03	1.00	0.99	7.75	4.92	0.13	0.20
	15	2.37	2.15	0.74	0.30	0.99	0.98	0.97	1.48	1.03	0.68
	25	2.41	2.64	1.60	8.35	1.00	0.99	0.71	0.39	1.41	2.57
	50	3.32	4.16	51.37	501.70	0.97	1.00	0.38	0.21	2.62	4.87
50PBS/50POM	5	1.71	1.93	0.02	0.01	1.00	1.00	8.74	8.99	0.11	0.11
	15	2.13	2.04	0.72	0.07	1.00	0.99	0.98	3.08	1.02	0.33
	25	1.94	2.73	1.16	4.06	1.00	0.99	0.77	0.52	1.30	1.91
	50	1.68	3.20	1.15	73.10	0.98	1.00	0.74	0.23	1.35	4.29
50PBS/50POM/ 0.5MWCNT	5	1.61	1.91	0.01	0.02	0.99	0.96	20.38	6.40	0.05	0.16
	15	2.18	2.26	0.22	0.17	1.00	0.98	1.69	1.86	0.59	0.54
	25	2.05	2.08	0.96	4.42	1.00	0.96	0.85	0.41	1.17	2.44
	50	2.14	2.88	1.20	58.50	1.00	1.00	0.77	0.21	1.29	4.67
50PBS/50POM/ 1MWCNT	5	1.54	1.61	0.01	0.04	0.97	1.00	15.68	5.88	0.06	0.17
	15	2.10	1.71	0.20	0.16	1.00	1.00	1.81	2.36	0.55	0.42
	25	1.59	2.58	0.12	6.39	0.97	0.94	3.01	0.42	0.33	2.37
	50	2.48	2.50	1.55	6.19	0.93	0.93	0.74	0.42	1.36	2.40

**Table 10:** Combined Avrami–Ozawa kinetic parameters of PBS, POM, PBS/MWCNT and POM/MWCNT nanocomposites.

Sample	X(t) (%)	α	F(T)	R^2^
PBS	10	1.76	3.49	0.99
	30	1.73	6.30	0.99
	50	1.73	8.29	1.00
	70	1.73	10.46	0.99
	90	1.75	13.57	0.99
PBS/0.5MWCNT	10	1.42	2.20	0.97
	30	1.48	3.32	0.96
	50	1.53	4.35	0.96
	70	1.58	5.66	0.96
	90	1.68	7.83	0.95
PBS/1MWCNT	10	1.23	3.28	0.95
	30	1.39	5.90	0.94
	50	1.53	7.46	0.94
	70	1.70	9.89	0.94
	90	1.89	15.22	0.94
POM	10	1.87	1.41	0.94
	30	2.47	2.30	0.96
	50	2.73	4.33	0.97
	70	2.91	7.95	0.99
	90	2.93	17.85	1.00
POM/0.5MWCNT	10	1.87	1.25	0.92
	30	1.94	3.09	0.92
	50	1.99	5.59	0.90
	70	2.05	9.07	0.90
	90	2.21	15.53	0.91
POM/1MWCNT	10	1.36	2.72	0.96
	30	1.81	3.50	0.95
	50	2.17	4.95	0.97
	70	2.40	7.95	0.98
	90	2.64	15.94	0.99

**Table 11:** Combined Avrami–Ozawa kinetic parameters of PBS/POM blends and PBS/POM/MWCNT nanocomposites.

Sample	X(t) (%)	α		F(T)		R^2^	
		PBS	POM	PBS	POM	PBS	POM
75PBS/25POM	10	1.00	1.11	6.56	4.38	0.98	0.97
	30	1.06	1.20	8.25	5.72	0.98	0.98
	50	1.12	1.28	9.61	6.88	0.98	0.98
	70	1.19	1.36	11.09	8.18	0.99	0.99
	90	1.25	1.48	13.75	10.53	0.99	0.99
75PBS/25POM/ 0.5MWCNT	10	1.14	1.22	5.70	2.38	0.99	1.00
	30	1.14	1.42	8.10	2.94	0.98	0.99
	50	1.15	1.52	9.58	3.82	0.98	1.00
	70	1.18	1.63	11.09	4.94	0.99	1.00
	90	1.24	1.76	13.34	7.49	0.99	1.00
75PBS/25POM/ 1MWCNT	10	1.13	1.79	5.66	1.05	0.97	0.96
	30	1.16	2.18	7.71	1.44	0.96	0.97
	50	1.22	2.37	9.17	2.12	0.96	0.98
	70	1.27	2.54	10.58	3.17	0.96	0.98
	90	1.39	2.76	13.19	5.72	0.96	0.98
50PBS/50POM	10	0.48	1.62	7.31	1.91	0.83	1.00
	30	0.48	1.83	9.33	2.95	0.86	1.00
	50	0.46	2.06	11.09	4.10	0.86	1.00
	70	0.47	2.20	12.47	5.93	0.87	0.99
	90	0.47	2.39	13.67	9.68	0.87	0.98
50PBS/50POM/ 0.5MWCNT	10	0.93	1.50	7.54	1.50	1.00	0.94
	30	0.96	1.72	11.54	2.15	1.00	0.93
	50	0.97	1.98	14.84	2.86	1.00	0.89
	70	0.96	2.05	18.14	4.46	1.00	0.92
	90	0.96	2.20	22.51	7.72	1.00	0.95
50PBS/50POM/ 1MWCNT	10	1.14	1.99	6.97	1.31	1.00	0.88
	30	1.12	2.17	12.11	1.59	1.00	0.87
	50	1.08	2.34	15.77	2.57	0.98	0.88
	70	1.05	2.42	20.75	4.53	0.99	0.89
	90	1.04	2.55	26.22	8.59	1.00	0.90

## Data Availability

The data presented in this study are available on request from the corresponding author.
